# Application of SCM with Bayesian B-Spline to Spatio-Temporal Analysis of Hypertension in China

**DOI:** 10.3390/ijerph15010055

**Published:** 2018-01-02

**Authors:** Zirong Ye, Li Xu, Zi Zhou, Yafei Wu, Ya Fang

**Affiliations:** 1 State Key Laboratory of Molecular Vaccinology and Molecular Diagnostics, School of Public Health, Xiamen University, Xiang’an Nan Road, Xiang’an District, Xiamen 361102, Fujian, China; yezirong0907@163.com (Z.Y.); zhouzi@xmu.edu.cn (Z.Z.); wyfyyahcx@163.com (Y.W.); 2 Key Laboratory of Health Technology Assessment of Fujian Province University, School of Public Health, Xiamen University, Xiang’an Nan Road, Xiang’an District, Xiamen 361102, Fujian, China; 3 Department of Statistics, School of Economics and Trade, Guangdong University of Foreign Studies, Guangzhou 510006, Guangdong, China; xuli85@126.com

**Keywords:** spatial analysis, disease mapping, hypertension, Shared Components Model (SCM), Bayesian B-spline

## Abstract

Most previous research on the disparities of hypertension risk has neither simultaneously explored the spatio-temporal disparities nor considered the spatial information contained in the samples, thus the estimated results may be unreliable. Our study was based on the China Health and Nutrition Survey (CHNS), including residents over 12 years old in seven provinces from 1991 to 2011. Bayesian B-spline was used in the extended shared component model (SCM) for fitting temporal-related variation to explore spatio-temporal distribution in the odds ratio (OR) of hypertension, reveal gender variation, and explore latent risk factors. Our results revealed that the prevalence of hypertension increased from 14.09% in 1991 to 32.37% in 2011, with men experiencing a more obvious change than women. From a spatial perspective, a standardized prevalence ratio (SPR) remaining at a high level was found in Henan and Shandong for both men and women. Meanwhile, before 1997, the temporal distribution of hypertension risk for both men and women remained low. After that, notably since 2004, the OR of hypertension in each province increased to a relatively high level, especially in Northern China. Notably, the OR of hypertension in Shandong and Jiangsu, which was over 1.2, continuously stood out after 2004 for males, while that in Shandong and Guangxi was relatively high for females. The findings suggested that obvious spatial–temporal patterns for hypertension exist in the regions under research and this pattern was quite different between men and women.

## 1. Introduction

Hypertension is a common chronic disease and a main risk factor for cardiovascular and cerebrovascular diseases. According to the 2013 World Health Organization (WHO) report, 9.4 million people were dying of complications due to hypertension. The continuous worldwide high prevalence of hypertension, especially in low- and middle-income countries, severely consumes medical and social resources, placing a heavy burden on families and countries. Using data from the 2010 National Population Census, the total number of people with hypertension in China was approximately 270 million. The Survey on the Status of Nutrition and Health of the Chinese People in 2012 showed that 25.2% of adults aged 18 or older in China had hypertension [1]. Inhabitants in China aged 18 or older have been reported to have a hypertension prevalence of 33.5%, with the rate in urban areas higher than that of rural areas (34.7% vs. 32.9%); the prevalence in men was higher than that in women (35.1% vs. 31.8%), and the prevalence in Eastern China, central China, and Western China were 36.2%, 34.1%, and 28.8%, respectively, in 2010 [2]. This indicated that the prevalence of hypertension in China has significant spatio-temporal variations in addition to being continuously high, providing a new direction for research into the risk factors for hypertension and its preventive control.

To explore spatial variations of diseases, spatial epidemiology is a systematic branch of epidemiology [3]. Disease mapping, especially Bayesian disease mapping, is a research hotspot in the field of spatial epidemiology. It visually identifies spatial patterns of disease and presents the results from a spatial perspective [4]. Furthermore, presenting result on a map can allow the acquisition of an intuitive and perceptual understanding, providing clues for further etiology research. In addition, disease mapping provides a rapid and visualized summary of complicated geographic information, enabling the identification of subtle patterns not visible in tabular presentations [5]. With advances in computer technology, such as the Markov chain Monte Carlo (MCMC) method, the applications of the Bayesian hierarchical models (BHM) have become increasingly popular due to its flexibility and easy expansion. Gelman acknowledged that BHM is a flexible tool for combining information and the partial pooling of inferences, providing a more objective method for inferences by estimating the parameters of prior distributions from data rather than requesting the data to be specified using subjective information [6]. 

The shared components model (SCM) is a widely-used disease mapping method based on the BHM framework. SCM was introduced by Knorr and Best [7]. SCM has been attracting increasing amounts of attention in the research on Bayesian joint disease mapping of two diseases or multiple diseases. It operates on the basic idea that many diseases share common risk factors, thus the underlying risk variations can be decomposed into shared and disease-specific components. The shared components, also called unobservable covariates, are the common risk factors of multiple diseases, whereas the disease-specific components represent space-varying disease-specific risk factors. SCM was used in many studies, such as for gender correlation of disease risk, sparse data in small areas, errors occurring in covariates, disease risk estimates for data from multiple sources, omission of estimated date, and disability-adjusted life year (DALY) in small areas [8,9,10,11,12,13,14,15,16,17].

According to these studies, the SCM has become an increasingly popular model for disease mapping due to its flexibility, convenience, and robustness of estimates. However, simultaneously including the spatial and temporal dependence structures in a model is difficult; previous research has mainly focused on spatial analysis. However, these studies were mainly conducted to explore the morbidity or mortality risk of rare diseases, such as cancer, in limited areas, whereas studies on non-rare disease were rarer. 

Thus, we tried to incorporate time trends into SCM using Bayesian B-spline fitting. This study explored the hypertension prevalence pattern and the evolution of its relevant factors, in seven provinces in China from 1991 to 2011, according to China Health and Nutrition Survey (CHNS) data. Considering that hypertension is a chronic disease, some lifestyles, such as smoking and drinking alcohol, may have a lagging effect. We performed an overall analysis combining the risk estimate with a population index. As the time span of this study is quite long (20 years), which may generate spatio-temporal interactions, we introduced interactive items into the model with the goal of better exploring the spatio-temporal evolution patterns in hypertension risk, thus providing guidance for spatio-temporal monitoring of diseases and implementing appropriate health policies.

## 2. Materials and Methods

### 2.1. Data

We obtained the data from the China Health and Nutrition Survey (CHNS) program. The program, an ongoing open cohort and international collaborative project between the Carolina Population Center at the University of North Carolina at Chapel Hill and the National Institute for Nutrition and Health (NINH, former National Institute of Nutrition and Food Safety) at the Chinese Center for Disease Control and Prevention (CCDC), was designed to examine the effects of health, nutrition, and family planning policies and programs implemented by national and local governments and to determine how the social and economic transformation of Chinese society is affecting the health and nutritional status of its population. The survey has been conducted nine times (1989, 1991, 1993, 1997, 2000, 2004, 2006, 2009, and 2011), and mainly covered Liaoning, Heilongjiang, Jiangsu, Shandong, Henan, Hubei, Hunan, Guangxi, and Guizhou provinces. Liaoning was not covered in 1997, and Heilongjiang was not covered in 1991 or 1993, but the survey added a few samples from Beijing, Shanghai, and Chongqing in 2011. Considering the completeness of the data, we chose samples from seven provinces surveyed continuously from 1991 to 2011: Jiangsu, Shandong, Henan, Hubei, Hunan, Guangxi, and Guizhou. Furthermore, the provinces surveyed were categorized as follows: northern China (Jiangsu, Shandong and Henan), central China (Hubei and Hunan), and southern China (Guangxi and Guizhou). Details of the survey procedure are available at http://www.cpc.unc.edu/projects/china.

Blood pressure was measured by a physician using a standard mercury sphygmomanometer and it was measured after 10 min of rest, and then measured again after 30 s. The procedure was repeated three times to obtain the average for the final blood pressure. According to the WHO criteria, hypertension is defined as a systolic blood pressure greater than or equal to 140 mmHg, a diastolic blood pressure greater than or equal to 90 mmHg, or those who currently using antihypertensive drugs.

### 2.2. Statistical Methods

**Figure 1:**
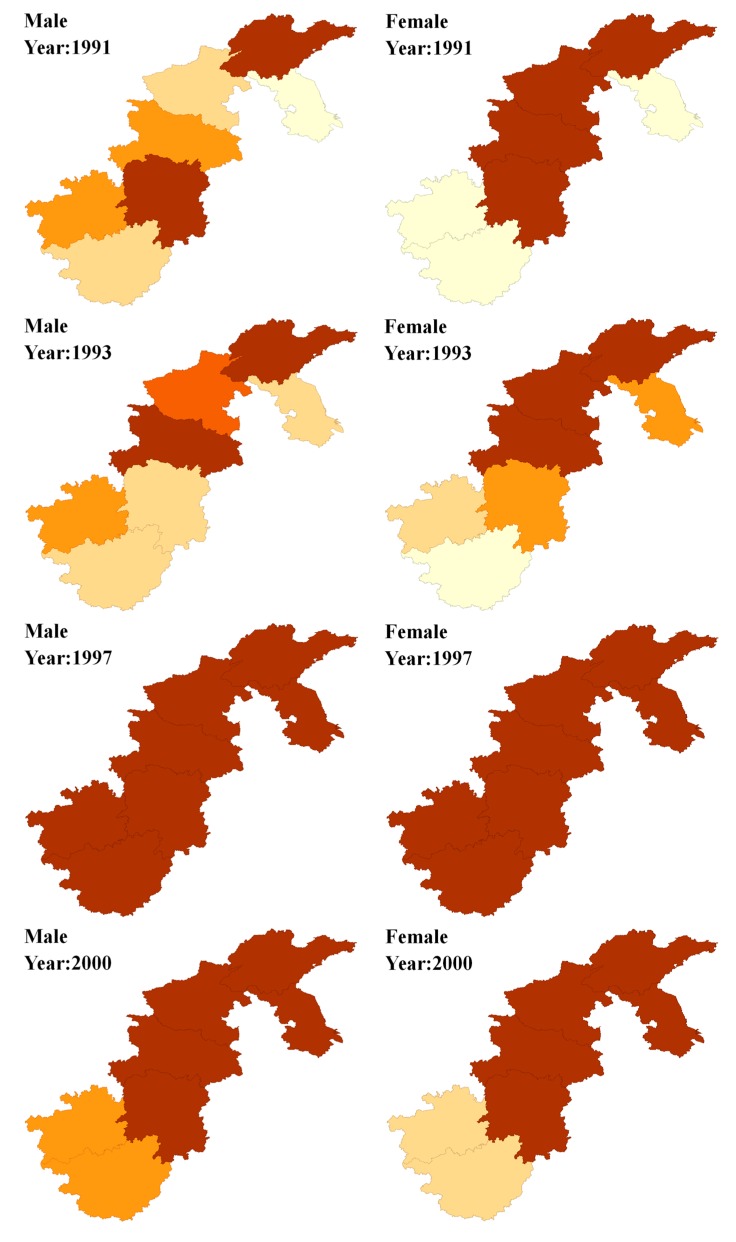

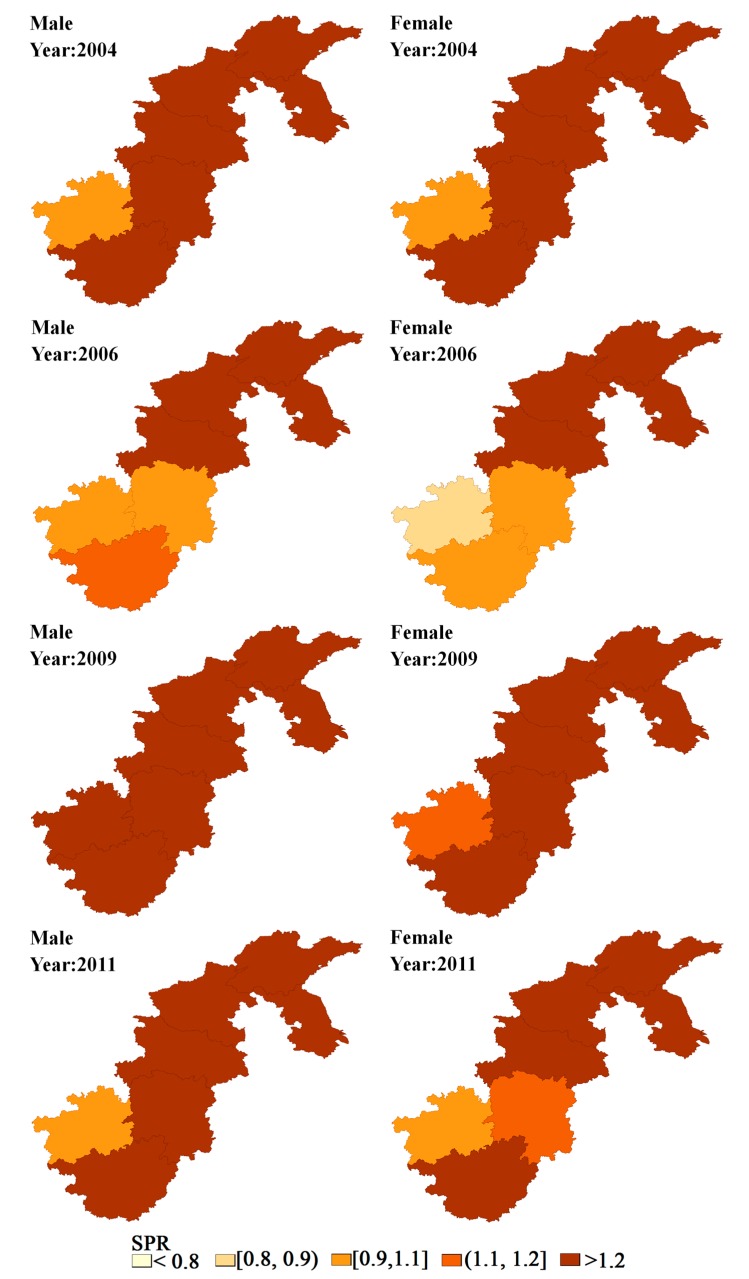
Maps of standardized prevalence ratios (SPRs) for male and female in each period.

The prevalence of hypertension was mapped to describe spatial distribution. Expected counts *E_jit_* were calculated for each year and province by the age-specific (four-year age groups) prevalence for these seven provinces in 1991 as the reference rates and using these to the annual population estimates for each area. Figure 1 shows the spatial distributions of the SPRs (SPR*_jit_* = *Y_jit_*/*E_jit_*) for the seven provinces during eight waves of surveys from 1991 to 2011. Furthermore, we performed Moran’s *I* to map the clustering of hypertension prevalence across the seven provinces. Moran’s *I* ranges from −1.0, completely dispersed, to +1.0, meaning completely clustered. *p* values were calculated as outputs along with Moran’s *I* [18].

### 2.3. Model

The study of SCM can be seen in Knorr and Best, Held et al., and Ibanez Beroiz et al. [19,20,21]. However, the models used in these studies only considered the spatial dimension. Moreover, these studies mainly focused on rare diseases, thus the corresponding Poisson distribution was used. Therefore, our study broadened the existing SCM to include both the spatial and temporal dimensions by adding the Bayesian B-spline, and extended its application to hypertension, a non-rare disease.

In this section, we constructed our model within the BHM framework. *E_jit_* and *n_jit_* represents the expected counts of cases and total number of observations for gender *j* (*j* = 1, 2), region *i* (*i* = 1, 2,…7) and time period *t* (*t* = 1, 2,…8). Let *p_jit_* represent the underlying disease rate for the given time and area.

$$E_{jit}\sim bin\left(n_{jit},p_{jit}\right)$$

$$logit\left(p_{jit}\right)=\alpha_{j}+eta_{jit}$$

where *α_j_* is the gender-specific intercept and represents the logarithm of the odds of the baseline of the entire region under research. The space trend, time trend, and space-time trend were explained on the logit scale by the joint structure of *eta_jit_*. We should refer to *exp*(*eta_jit_*) (*exp*(*eta_jit_*) = (*p_jit_*/(*1* − *p_jit_*))/*exp*(*α_j_*)) as a relative odds ratio (OR), which is a ratio of the specific time and area odds over the baseline odds.

We assumed that a correlation existed between men and women for hypertension, so we applied joint disease mapping:

$$eta_{1it}=\left(b_{0i}+RS_{0i}\left(t\right)\right)*\delta_{t}+S_{1}\left(t\right)+b_{1i}+RS_{1i}\left(t\right)$$

$$eta_{2it}=\frac{\left(b_{0i}+RS_{0i}\left(t\right)\right)}{\delta_{t}}+S_{2}\left(t\right)+b_{2i}+RS_{2i}\left(t\right)+\beta_{it}$$

$$\eta_{1}\left(t\right)=var\left(\left(b_{0i}+RS_{0i}\left(t\right)\right)*\delta_{t}\right)/var\left(eta_{1it}\right)$$

$$\eta_{2}\left(t\right)=var\left(\left(b_{0i}+RS_{0i}\left(t\right)\right)/\delta_{t}\right)/var\left(eta_{2it}\right)$$

where *b*_0*i*_ is the shared spatial variation for both genders; *RS*_0*i*_(*t*) is the shared spatio-temporal variation for both genders, so (*b*_0*i*_ + *RS*_0*i*_(*t*)) represents the overall common variation for both genders; *S_j_*(*t*) is the gender-specific temporal variation in the relative hypertension risk; *b_ji_* is the gender-specific spatial variation; *RS_ji_*(*t*) is the gender-specific spatio-temporal variation; *β_it_* represents the differential spatial pattern of women with respect to men for the individual year; *δ_t_* and 1/*δ_t_* represents the weights of the shared component for both genders in year *t*; and *η_j_*(*t*) represents the proportion variance shared for both genders in year *t*.

Although B-spline, smoothing spline, and P-spline are mainstream, MacNab suggested that P-spline and the smoothing spline mostly obtain reliable results with large samples [22]. Also, both methods are sensitive to hyperprior specification and are uncertain when the volume of the sample is limited. In contrast, B-spline can fit the sample preferably even when the sample is relatively small. Considering the limited research regions and time periods, to ensure the smoothness of the curve, the cubic B-spline function was selected to improve the smoothness of the fitting curve with L knots. Then, *RS*_0*i*_(*t*), *S_j_*(*t*), and *RS_ji_*(*t*) can be written as follows:

$$RS_{0i}\left(t\right)=\overset{K}{\underset{k=1}{\sum }}b_{ik}B_{k}\left(t\right)$$

$$S_{j}\left(t\right)=\overset{K}{\underset{k=1}{\sum }}a_{jk}B_{k}\left(t\right)$$

$$RS_{ji}\left(t\right)=\overset{K}{\underset{k=1}{\sum }}\beta_{jik}B_{k}\left(t\right)$$

where *a_jk_* = (*j* = 1, 2; *k* = 1,…*k*) is the coefficient of the fixed spline, *B_k_* (*k* = 1,…*k*) are a series of basic functions, *B_k_*(*t*) represents the *k*th B-spline functions at time *t* (*K* = *L* + 3); *b_k_*(*b*_1*k*_,…,*b_nk_*)*^T^*, and *β_jk_(β_j1k_,…,β_jNk_)^T^* are the coefficients of random splines. To ensure the identifiability of the model, the basic function *B_k_* did not include an intercept. By explorative analysis, we initially set a knot at year 1997, and then added a knot in both 2000 and 2004. According to the coefficients of the spline, we deleted *RS*_0*i*_(*t*) and *RS_ji_*(*t*) from the initial model to reduce the complexity of the model and increase the identifiability. As a result, the seven models constructed in this part can be seen in Table 1.

For prior distributions, N (0.0, 10) distribution was assigned for the logarithm of weight *δ_t_*; CAR (conditional autoregressive) was assigned for *β_it_* and *β_jk_*; N (0, 10,000) was assigned for *a_jk_*, *b_ji_*, and *b*_0*i*_; and MCAR (multivariate conditional autoregressive regression) for the coefficient of random spline *b_k_*. To increase the identifiability and decrease the complexity of the models, unstructured normal priors were assumed for *b*_0*i*_ and *b_ji_*.

All the above models were fitted using the Markov chain Monte Carlo (MCMC) method. For reliability and comparison between models, two independent MCMC chains were run for each model with a total of 50,000 iterations, after a 5000-iteration burn-in period for both chains. Graphical checks of chains, as well as their autocorrelations and Brooks–Gelman–Rubin diagnostics, were performed to assess convergence. After convergence, the commonly used deviance information criterion (DIC) was calculated to guide model selection. Finally, to assess the sensitivity of the different models, the precision parameters were set for different prior distributions [23].

Data extraction, management, and description were performed in SAS (9.4, SAS Institute lnc., Cary, NC, USA). The SCM models were run in the OpenBUGS 3.2.3 software. Maps of these provinces were produced in GeoDa 1.8.16.4. The OpenBUGS codes used for these models are available as Supplementary Materials File 1.

## 3. Results

### 3.1. Statistical Descriptions

The samples of the different provinces in different years are shown in Table 2 and Table 3. In addition, we used 12–29 samples as reference group; the remaining population was divided into groups based on age: the young group aged 30–44, the middle-aged group (45–59), and the elderly group aged 60 and older, according to WHO criteria.

**Figure 2:**
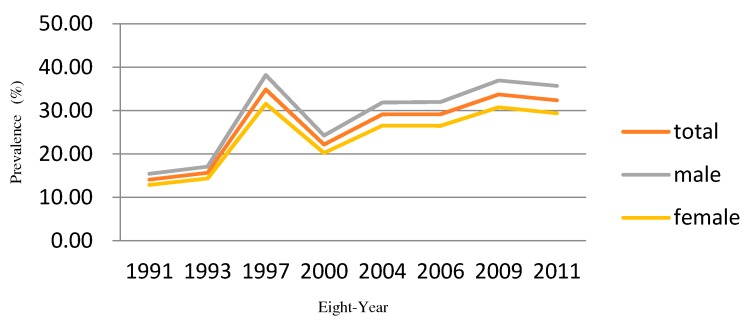
Prevalence of hypertension during eight waves of surveys from 1991 to 2011.

The variations in hypertension prevalence between men and women were analyzed based on the data from the seven provinces. The study used the survey data obtained through eight waves of follow-ups, from 1991 to 2011. An overall collective upward trend in the prevalence of hypertension was present. The overall prevalence for these provinces was 25.4%, with men experiencing a higher prevalence than women (27.9% vs. 23.0%). The prevalence for men was significantly higher than for women in each year, whereas both showed a similar upward trend over time. The evolution could be roughly divided into two phases, with the year 2000 as a boundary point. Before 2000, except for 1997, both men and women had a relatively low prevalence of less than 17%. After 2000, both gradually increased to 30% or above. Notably, an abrupt increase could be seen in the prevalence of hypertension for both men and women in 1997, increasing by 1.2 times compared to the prior year (Figure 2). More details about prevalence of hypertension during eight waves of surveys of each region can be seen in Supplementary Materials File 2.

To further investigate the spatial patterns of hypertension risk, we examined the SPR levels of the seven provinces from 1991 to 2011. The spatial distributions of the SPR for men and women from 1991 to 2011 are shown in Figure 1. Overall, the SPR for men and women older than 12 were a little higher in Northern China than in Southern China, with a more evident time trend for men than women. Before 1997, the SPR of hypertension for men in the Henan province had an evident upward trend over time and remained at a relatively high level after that, whereas it was more stable for women. Furthermore, geographic and gender variations in SPR differed slightly in different years. Among these, the spatial distribution of hypertension in the SPR for men and women were nearly identical in 1997, 2000, 2004 and 2006, whereas the gender variation existed in other years. Also, the distribution of hypertension in the SPR showed evident spatial patterns from 1991 to 2011, except in 1997. For men, the SPR in Shandong province basically remained at a relatively high level of more than 1.2 during the period, with Henan province continually ranking first after 2000. As for women, the SPR in Henan province remained at the highest level for multiple years, with Shandong, Guangxi and Hubei ranking first in only a few years of the study.

The Moran’s *I* values can be seen in Table 4. Hypertension prevalence in these provinces was significantly clustered in 1991 (Moran’s *I* = −0.0911, *p* = 0.037), 1993 (Moran’s *I* = 0.5002, *p* = 0.042), 2004 (Moran’s *I* = 0.3986, *p* = 0.044), 2006 (Moran’s *I* = 0.4739, *p* = 0.047), and 2011 (Moran’s *I* = 0.6952, *p* = 0.012). We found that a global cluster of hypertension prevalence occurred in most years. Thus, further geographical study is necessary.

### 3.2. Model Comparison and Fitting

The model comparison criteria of these models are presented in Table 5. These criteria include Dbar, pD and DIC.

As too many components in the model might lead to an identifiability problem, the model was built to perform analysis using 1997 as a boundary point. The results showed that the random spline coefficient *β_jk_* was close to zero, indicating that no evident gender-specific spatio-temporal variation existed, so the random splines were removed from the model. The next step was to further examine whether a similar time trend existed for the hypertension risk for men and women. The results showed that the DIC of the model without *RS*_0*i*_(*t*) was considerably smaller, implying that no evident spatio-temporal variations were common to men and women. Hence, for the sake of brevity, *RS*_0*i*_(*t*) and *RS_ji_*(*t*) were removed from the model. From Table 5, when the number of knots was two, the fitting effects of the model were the best (smallest Dbar). In comprehensively analyzing all the factors, such as Dbar, pD and DIC of the model, we chose Model 5 as the final model.

### 3.3. Results of the Model

**Figure 3:**
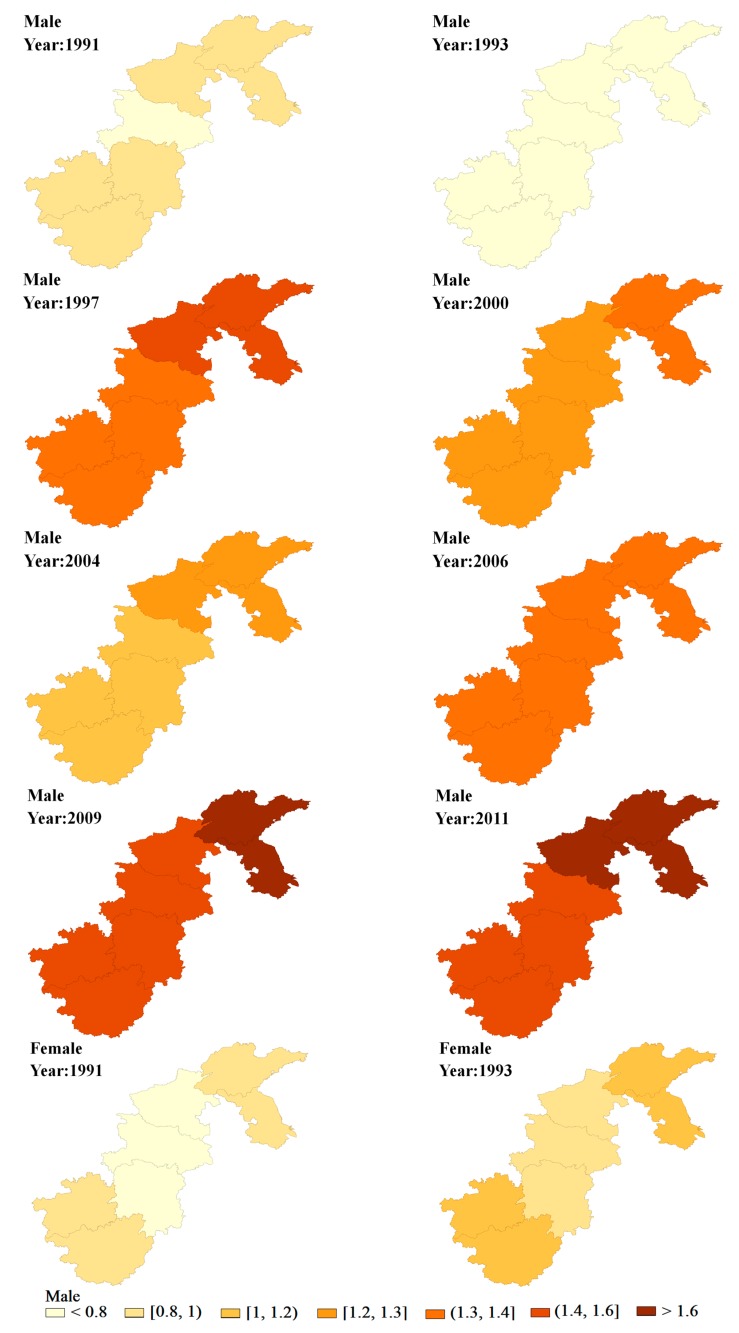

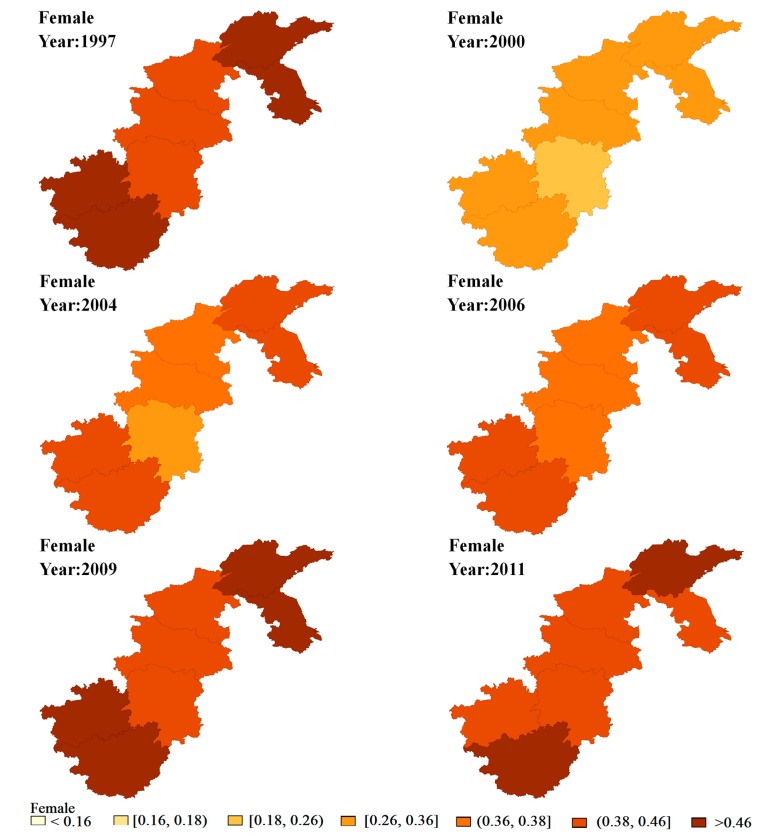
Maps of the odds ratio (OR) for shared component model (SCM) in each period.

Based on the above comparisons, we present the results of Model 5. Figure 3 shows the spatial distribution of the hypertension risk in the seven provinces in China from 1991 to 2011, derived from the SCM spatio-temporal model. Spatial variations obviously existed between the hypertension risk of men and women after the smoothing of the SCM model. Both men and women had a relatively high hypertension risk in Northern China and both had a relatively low hypertension risk in Hunan province. For women, Shandong and Guangxi showed a relatively high risk, whereas Shandong and Jiangsu had a relatively high risk for men. In terms of the time trend, on the condition that the mapping split points were the same, the colors in multiple provinces for men and women became darker and darker over time, in accordance with the overall upward trend of hypertension prevalence rates presented in Figure 2.

**Figure 4:**
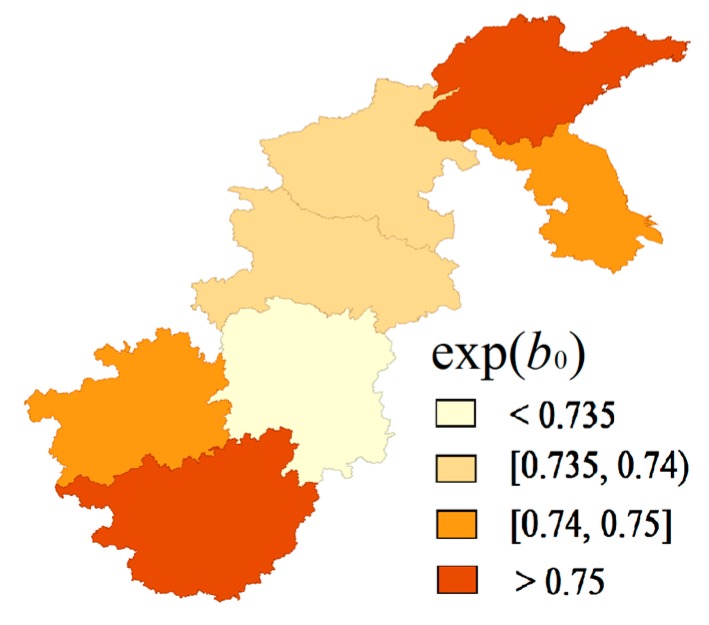
Maps of the exp(*b_0_*) for shared component model (SCM).

**Figure 5:**
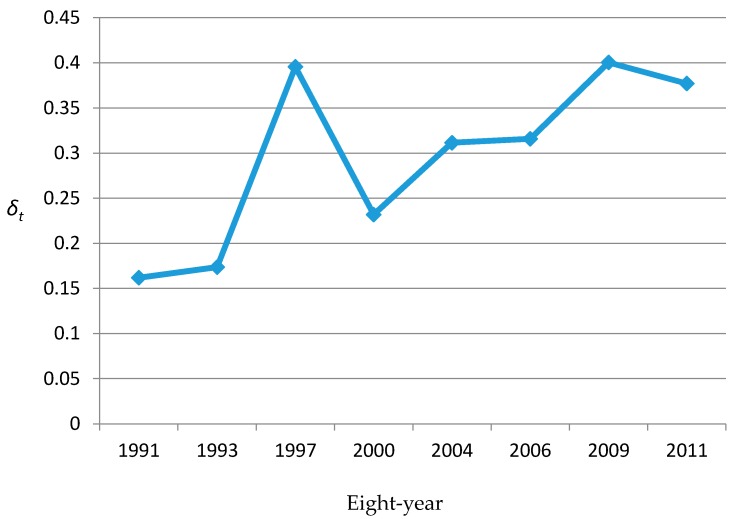
Weights *δ_t_* of SCM during eight waves surveys (from 1991 to 2011).

Figure 4 shows the common spatial variations derived from the extended SCM model. These patterns were clearly characterized by looking separately at the spatial distribution of shared components. From the spatial shared components viewpoint, the high prevalence that resulted from shared components could be seen in Shandong and Guangxi, whereas the rates in other provinces were relatively low, especially in Hunan. We further calculated that gender variation (*β_it_*) which showed no evident spatial variation and its values for different years mainly ranged from 0.998 to 1.002. Figure 5 shows that the posterior mean of *δ* in each year was smaller than one. As for the time trend, the posterior mean of *δ* in each year steadily increased over time, except in 1997, with an abrupt increase, which aligns with the time trends of the hypertension prevalence rates in most provinces, especially for Shandong and Henan, in the descriptive analysis.

### 3.4. Convergence and Sensitivity Analysis of SCM Modeling

**Figure 6:**
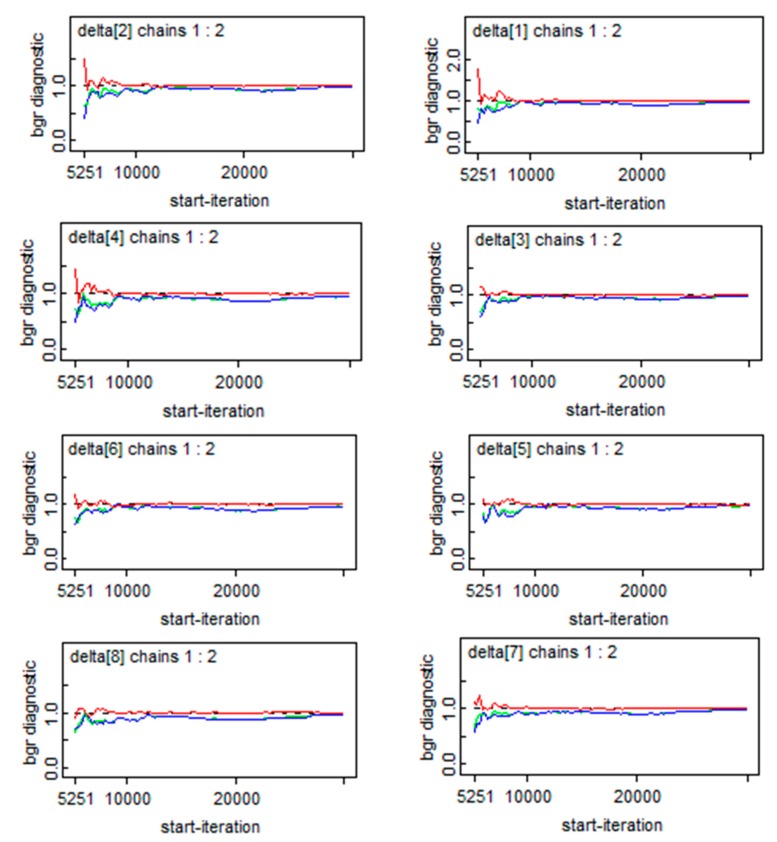
Convergence of *δ_t_*.

To insure the reliability of the model, the convergence of the parameters were diagnosed and the sensitivity of the model to the priors chosen for the precision parameters was also assessed. Figure 6 briefly describes the convergence of the essential parameters of the *δ_t_*, which illustrated that convergence was achieved. As for the sensitivity analysis, we set three prior distributions: priors1: *τ_β_*~gamma (5.0,5.0 × 10^−4^); priors 2: *σ_β_*~ unif (0,1); priors3: *σ^2^_β_*~ norm (0.0,0.01) *I* (0,∞). Table 6 showed the results of the sensitivity analysis, which verified that the posterior estimates of these parameters and the resulting DIC and pD were both robust in regard to the moderate modification in the prior distributions, thus the final model presented above was appropriate.

## 4. Discussion

The high prevalence of hypertension not only affects quality of life but also creates a heavier disease burden for both individuals and society as a whole. To advance the efficiency of disease control, specialists tried to identify the areas with high hypertension prevalence and reveal the hypertension risk factors. Previous research found that gender variations may exist in hypertension prevalence [24,25,26]; however, the research was limited because most of the studies were conducted using traditional statistical methods, such as logistic regression, which do not consider spatial information. Hence, the mixture of lifestyle choices and environmental factors, which might cause the spatial variation in the risk of hypertension, was not effectively revealed. As for this study, SCM was performed to explore regional gender variation in the hypertension risk in seven provinces of China. This paper focused on hypertension, a common disease, and broadened the exiting SCM by adding Bayesian B-spline to fit time-related random components. As a result, these models could be applied to investigate regional and gender variations in hypertension risk, and whether the spatial distribution of hypertension risk is formed by persistence or chance, and to study non-rare diseases to explore risk factors at the regional level.

Given the results derived from the spatio-temporal analysis, the spatio-temporal distribution of hypertension exhibited certain spatial clustering. The prevalence was higher in the north, such as in Jiangsu, Shandong and Henan, which was the same as found in previous research [27]. Moreover, the boundary points of the time trend in the hypertension risk occurred in 1997, 2000, and 2004 for men, whereas it occurred in 1997 and 2004 for women. Before 1997, the hypertension risk for both genders remained low. After that, especially since 2004, the hypertension risk in all seven provinces increased over time, particularly in northern China, in some neighboring provinces, such as Jiangsu, Shandong and Henan. However, in the neighboring provinces, such as Hunan and Guizhou, the hypertension risk remained relatively low. Notably, the hypertension risk in Shandong and Jiangsu province, for men in particular, continuously stood out after 2004, so special attention should be paid to this region for hypertension prevention and control.

Further analysis of the above-mentioned spatial–temporal variations showed that the prevalence of hypertension in provinces like Jiangsu, Shandong, Henan provinces remained relatively high, whereas the rates in Hunan and Guizhou remained at an evidently lower level. Given the fact that the proportions of obesity and alcohol consumption are high in the northern provinces, the above findings may indicate that factors associated with lifestyle, such as obesity [28,29] and alcohol consumption [30], might be the main causes of the increasing hypertension risk in some regions. Furthermore, the inhabitants in provinces with a high hypertension risk prefer foods with stronger flavors in their daily life, and high-salt diets were quite typical. Therefore, high-salt diets might be another important reason for the increasing hypertension risk, verifying most of the research conclusions [31,32]. Thus, to reduce the prevalence of hypertension in northern China, some intervention programs aimed at lifestyle choices should be implemented. 

To be specific, from 2004 onwards, the hypertension risk for men in Henan remained at a relatively high level. Further analysis found that the smoking rate for men in Henan was considerably higher than that in other provinces prior to 2004, implying that the negative influence of smoking on hypertension might have a time lag of at least 10 years, consistent with previous research [33,34]. 

The distribution of *β_it_* indicated that the hypertension risk presented no evident gender variation and remained stable over time after removing the spatio-temporal variations common and specific to both genders. The results of other SCM parameters are shown in Figure 4 and Figure 5. Combining the results of the common components and the corresponding weight (*δ*) may suggest that some common unobserved factors, such as atmosphere pollution, dominated the spatio-temporal variations in the risk of hypertension for men and women [35]. 

We believe our study will contribute to the medical literature in the following ways: (1) To broaden the existing SCM, Bayesian B-spline was added to fit the time trend so that spatial and temporal information could be simultaneously and effectively incorporated into the model. Therefore, the continuity and contingency patterns of disease risk were clearly shown, providing reliable information for the deeper exploration of disease causes. (2) Our study showed that, though often applied to rare diseases, the SCM can also be applied to non-rare diseases to identify risk factors at the regional level and quantify gender-specific fixed effects [36].

Limited by time and research conditions, some areas of this study need further study and improvements. Firstly, this study used the province level as the analysis scale, which might lead to considerable uncertainty for the statistical inference of parameters, but data on a finer scale, such as at the city level or the county level, could not be acquired due to some principled requests, such as the privacy of survey data. Secondly, in the spatial analysis of SCM, the commonly-used CAR and Gaussian distribution were assumed for the spatially structured and unstructured random effects, respectively. As a result, this may lead to not fully estimating the impact of some situations, such as different degrees of spatial correlations occurring and extreme values existing in samples on the estimates of disease risk, which is the future direction of this work. Thirdly, in this study, some hypertension risk factors, such as smoking and drinking, were not introduced into the spatio-temporal models as covariates, hence, a deeper explanation for the spatio-temporal variations of risk factors is lacking. However, the increase in complexity may lead to a failure of the identification of the SCM, which restricted our research to some extent.

## 5. Conclusions

These shared component models identified the spatial–temporal trend in hypertension risk for the populations in seven provinces in China, and provided evidence supporting the existence of obvious gender variations in the spatial–temporal pattern of hypertension risk. However, the differential spatial–temporal trends between men and women that might be linked to lagged uptake of smoking and other lifestyle choices also meant that current inequalities in these risk factors between areas have remained unclear, providing avenues for future research.

## Figures and Tables

**Table 1:** The seven constructed models.

Model No.	Characteristic
1	Knot at 1997, with *RS*_0*i*_(*t*)
2	Knot at 1997, without *RS*_0*i*_(*t*)
3	Knots at 2000, with *RS*_0*i*_(*t*)
4	Knots at 2000, without *RS*_0*i*_(*t*)
5	Knots at 1997 and 2000, without *RS*_0*i*_(*t*) and *RS_ji_*(*t*)
6	Knots at 1997 and 2004, without *RS*_0*i*_(*t*) and *RS_ji_*(*t*)
7	Knots at 1997, 2000 and 2004, without *RS*_0*i*_(*t*) and *RS_ji_*(*t*)

**Table 2:** Trends in prevalence of hypertension among the participants (%).

Province	Variable	1991	1993	1997	2000	2004	2006	2009	2011
Jiangsu	Number	1255	1213	1443	1277	1186	1131	1255	1178
	Cases	134	164	530	302	350	426	491	435
	Prevalence (%)	10.68	13.52	36.73	23.65	29.51	37.67	39.12	36.93
Shandong	Number	1195	1092	1380	1146	1111	1131	1142	1084
	Cases	249	234	532	345	361	336	377	388
	Prevalence (%)	20.84	21.43	38.55	30.10	32.49	29.71	33.01	35.79
Henan	Number	1124	1036	1498	1127	1338	1191	1239	1183
	Cases	166	192	599	303	564	500	489	488
	Prevalence (%)	14.77	18.53	39.99	26.89	42.15	41.98	39.47	41.25
Hubei	Number	1269	1191	1513	1167	1146	1049	1068	1015
	Cases	188	229	555	231	347	375	377	356
	Prevalence (%)	14.81	19.23	36.68	19.79	30.28	35.75	35.30	35.07
Hunan	Number	1259	1211	1405	1184	1142	1224	1196	1131
	Cases	193	155	443	290	265	242	341	317
	Prevalence (%)	15.33	12.80	31.53	24.49	23.20	19.77	28.51	28.03
Guangxi	Number	1362	1359	1688	1264	1356	1316	1443	1431
	Cases	172	173	619	201	353	293	471	380
	Prevalence (%)	12.63	12.73	36.67	15.90	26.03	22.26	32.64	26.55
Guizhou	Number	1549	1367	1687	1299	1216	1181	1138	1079
	Cases	168	180	424	204	232	223	315	258
	Prevalence (%)	10.85	13.17	25.13	15.70	19.08	18.88	27.68	23.91

**Table 3:** Age distribution of the participants.

Gender	Variable	Value	1991	1993	1997	2000	2004	2006	2009	2011
Male	Age	12–29	40.30	37.97	37.87	30.39	23.46	17.97	17.14	15.60
		30–44	27.84	28.39	27.17	26.59	25.55	27.59	25.50	22.45
		45–59	19.01	19.71	21.15	26.37	30.59	31.04	31.71	32.59
		60–100	12.86	13.92	13.80	16.65	20.40	23.40	25.65	29.35
Female	Age	12–29	39.31	35.13	34.33	26.16	19.43	15.59	15.46	14.22
		30–44	28.82	30.84	28.38	28.55	27.11	28.87	25.36	23.04
		45–59	18.39	19.91	20.88	26.51	31.02	30.49	31.86	32.70
		60–100	13.48	14.12	16.41	18.78	22.43	25.05	27.32	30.04

**Table 4:** Global cluster for prevalence of hypertension during eight waves of surveys.

Year	Moran’s *I*	*p*-Value
1991	–0.0911	0.037
1993	0.5002	0.042
1997	0.0344	0.227
2000	0.1270	0.216
2004	0.3986	0.044
2006	0.4739	0.047
2009	0.0616	0.269
2011	0.6925	0.012

**Table 5:** Result of the models including Dbar, pD, deviance information criterion (DIC).

Model	Dbar	pD	DIC
1	1569.0	11.82	1581.0
2	133.7.0	24.9	412.2
3	1342.0	14.2	1356.0
4	1323.0	25.0	1348.0
5	1105.0	25.0	1130.0
6	1263.0	25.2	1288.0
7	1338.0	19.6	1357.0

**Table 6:** Sensitivity analysis with respect to different priors for the precision parameters.

Parameter	Priors 1	Priors 2	Priors 3
	Mean (95% CI)	Mean (95% CI)	Mean (95% CI)
Exp(*α_j_*)			
Males	0.340 (0.312–0.370)	0.340 (0.312–0.369)	0.340 (0.312–0.369)
Females	0.948 (0.891–1.007)	0.949 (0.892–1.009)	0.949 (0.892–1.009)
*δ_1_*	0.161 (0.141–0.182)	0.162 (0.141–0.184)	0.162 (0.143–0.182)
OR			
Males	0.841 (0.834–0.849)	0.841 (0.834–0.849)	0.841 (0.834–0.849)
Females	0.175 (0.153–0.196)	0.166 (0.153–0.197)	0.166 (0.153–0.197)
DIC(pD)	DIC = 1105 (pD = 25.0)	DIC = 1106 (pD = 26.2)	DIC = 1108 (pD = 26.1)

For brevity, this table mainly presented results of sensitivity for *δ* when *t* = 1 and OR when *t* = 1 and region *i* = 1, results of other *δ* and OR can be obtained upon request.

## Supplementary Materials

The following are available online at www.mdpi.com/1660-4601/15/1/55/s1, Supplementary Materials File 1: the OpenBUGS code for our model; Supplementary Materials File 2: Prevalence of hypertension during eight waves surveys of each region.
