# Supplementary material for: Application of SCM with Bayesian B-Spline to Spatio-Temporal Analysis of Hypertension in China
Source: Int J Environ Res Public Health. 2018 Jan 2;15(1):55. doi: 10.3390/ijerph15010055 (PMC5800154; doi:10.3390/ijerph15010055)
Supplement: Supplementary file 1 [file ijerph-15-00055-s001.zip › supplementary1.pdf]

```
#Model 1
model
	{
		for(j in 1:M) {
		for( i in 1:N) {
		O1[j,i]~ dbin(p1[j,i],n1[j,i])
		O2[j,i]~ dbin(p2[j,i],n2[j,i])
		logit(p1[j,i]) <-alpha[1]+eta1[j,i,1]
		logit(p2[j,i]) <-alpha[2]+eta2[j,i,2]

eta1[j,i,1]<-(b0[i]+(b[1,i]*B1[j]+b[2,i]*B2[j]+b[3,i]*B3[j]+b[4,i]*B4[j]))*delta[j]+a1[1]*B1[j]+a1[2]*B2[j]+a1[3]*B3[j]+a1[4]*B4[j]+b1[i]

eta2[j,i,2]<-(b0[i]+(b[1,i]*B1[j]+b[2,i]*B2[j]+b[3,i]*B3[j]+b[4,i]*B4[j]))/delta[j]+a2[1]*B1[j]+a2[2]*B2[j]+a2[3]*B3[j]+a2[4]*B4[j]+b2[i]+beta[j,i]
	  }
	 }
		 # weights for the spatial adjacency matrix
		   for(k in 1: sumNumNeigh){
		      weights[k]<-1
		   }

	# Spatial priors
		  b[1:4,1:N]~ mv.car(adj[],weights[],num[],omega[,])
		   omega[1:4,1:4]~ dwish(R[,],4)
		  for(j in 1:M) {
		  beta[j,1:N] ~ car.normal(adj[],weights[],num[],tau.beta)

		  }
	# other priors ,such as the intercepts,fixed effects,relative weight and variances
	   alpha[1]~dnorm(0.0,100)
		  OR.alpha[1]<-exp(alpha[1])
		  alpha[2]~dnorm(0.0,1000)
		  OR.alpha[2]<-exp(alpha[2])

		 sigma2.beta<-1/tau.beta

		 tau.beta~dgamma(5,0.0005)

		 for (P in 1:4){
		    a1[P]~dnorm(0.0,100)
		    OR.a1[P]<-exp(a1[P])
		    a2[P]~dnorm(0.0,1000)
		    OR.a2[P]<-exp(a2[P])
```

```
                }
        for( i in 1:N) {
                b0[i] ~ dnorm(0.0,100)
                OR.b0[i]<-exp(b0[i])
                b1[i] ~ dnorm(0.0,100)
                OR.b1[i]<-exp(b1[i])
                b2[i] ~ dnorm(0.0,1000)
                OR.b2[i]<-exp(b2[i])
                                }

    #scaling factor for relative strength of shared component for each disease
     for (j in 1:M){
        logdelta[j]~ dnorm(0.0,100)
        delta[j]<-exp(logdelta[j])
     #ratio(relative risk of disease 1 associated with shared component ) to disease 2 associated with shared component
           OORatio[j]<-pow(delta[j],2)
      }
#ouput
   for (i in 1:N){
      OR.beta1[i]<-exp(beta[1,i])
      OR.beta2[i]<-exp(beta[2,i])
      OR.beta3[i]<-exp(beta[3,i])
      OR.beta4[i]<-exp(beta[4,i])
      OR.beta5[i]<-exp(beta[5,i])
      OR.beta6[i]<-exp(beta[6,i])
      OR.beta7[i]<-exp(beta[7,i])
      OR.beta8[i]<-exp(beta[8,i])


OR11[i]<-exp( (b0[i]+(b[1,i]*B1[1]+b[2,i]*B2[1]+b[3,i]*B3[1]+b[4,i]*B4[1]))*delta[1]+a1[1]*B1[1]+
a1[2]*B2[1]+a1[3]*B3[1]+a1[4]*B4[1]+b1[i])

OR12[i]<-exp( (b0[i]+(b[1,i]*B1[2]+b[2,i]*B2[2]+b[3,i]*B3[2]+b[4,i]*B4[2]))*delta[2]+a1[1]*B1[2]+
a1[2]*B2[2]+a1[3]*B3[2]+a1[4]*B4[2]+b1[i])

OR13[i]<-exp( (b0[i]+(b[1,i]*B1[3]+b[2,i]*B2[3]+b[3,i]*B3[3]+b[4,i]*B4[3]))*delta[3]+a1[1]*B1[3]+
a1[2]*B2[3]+a1[3]*B3[3]+a1[4]*B4[3]+b1[i])

OR14[i]<-exp( (b0[i]+(b[1,i]*B1[4]+b[2,i]*B2[4]+b[3,i]*B3[4]+b[4,i]*B4[4]))*delta[4]+a1[1]*B1[4]+
a1[2]*B2[4]+a1[3]*B3[4]+a1[4]*B4[4]+b1[i])

OR15[i]<-exp( (b0[i]+(b[1,i]*B1[5]+b[2,i]*B2[5]+b[3,i]*B3[5]+b[4,i]*B4[5]))*delta[5]+a1[1]*B1[5]+
a1[2]*B2[5]+a1[3]*B3[5]+a1[4]*B4[5]+b1[i])
```

```
OR16[i]<-exp( (b0[i]+(b[1,i]*B1[6]+b[2,i]*B2[6]+b[3,i]*B3[6]+b[4,i]*B4[6]))*delta[6]+a1[1]*B1[6]+
a1[2]*B2[6]+a1[3]*B3[6]+a1[4]*B4[6]+b1[i])

OR17[i]<-exp( (b0[i]+(b[1,i]*B1[7]+b[2,i]*B2[7]+b[3,i]*B3[7]+b[4,i]*B4[7]))*delta[7]+a1[1]*B1[7]+
a1[2]*B2[7]+a1[3]*B3[7]+a1[4]*B4[7]+b1[i])

OR18[i]<-exp( (b0[i]+(b[1,i]*B1[8]+b[2,i]*B2[8]+b[3,i]*B3[8]+b[4,i]*B4[8]))*delta[8]+a1[1]*B1[8]+
a1[2]*B2[8]+a1[3]*B3[8]+a1[4]*B4[8]+b1[i])


OR21[i]<-exp( (b0[i]+(b[1,i]*B1[1]+b[2,i]*B2[1]+b[3,i]*B3[1]+b[4,i]*B4[1]))/delta[1]+a2[1]*B1[1]+
a2[2]*B2[1]+a2[3]*B3[1]+a2[4]*B4[1]+b2[i]+beta[1,i])

OR22[i]<-exp( (b0[i]+(b[1,i]*B1[2]+b[2,i]*B2[2]+b[3,i]*B3[2]+b[4,i]*B4[2]))/delta[2]+a2[1]*B1[2]+
a2[2]*B2[2]+a2[3]*B3[2]+a2[4]*B4[2]+b2[i]+beta[2,i])

OR23[i]<-exp( (b0[i]+(b[1,i]*B1[3]+b[2,i]*B2[3]+b[3,i]*B3[3]+b[4,i]*B4[3]))/delta[3]+a2[1]*B1[3]+
a2[2]*B2[3]+a2[3]*B3[3]+a2[4]*B4[3]+b2[i]+beta[3,i])

OR24[i]<-exp( (b0[i]+(b[1,i]*B1[4]+b[2,i]*B2[4]+b[3,i]*B3[4]+b[4,i]*B4[4]))/delta[4]+a2[1]*B1[4]+
a2[2]*B2[4]+a2[3]*B3[4]+a2[4]*B4[4]+b2[i]+beta[4,i])

OR25[i]<-exp( (b0[i]+(b[1,i]*B1[5]+b[2,i]*B2[5]+b[3,i]*B3[5]+b[4,i]*B4[5]))/delta[5]+a2[1]*B1[5]+
a2[2]*B2[5]+a2[3]*B3[5]+a2[4]*B4[5]+b2[i]+beta[5,i])

OR26[i]<-exp( (b0[i]+(b[1,i]*B1[6]+b[2,i]*B2[6]+b[3,i]*B3[6]+b[4,i]*B4[6]))/delta[6]+a2[1]*B1[6]+
a2[2]*B2[6]+a2[3]*B3[6]+a2[4]*B4[6]+b2[i]+beta[6,i])

OR27[i]<-exp( (b0[i]+(b[1,i]*B1[7]+b[2,i]*B2[7]+b[3,i]*B3[7]+b[4,i]*B4[7]))/delta[7]+a2[1]*B1[7]+
a2[2]*B2[7]+a2[3]*B3[7]+a2[4]*B4[7]+b2[i]+beta[7,i])

OR28[i]<-exp( (b0[i]+(b[1,i]*B1[8]+b[2,i]*B2[8]+b[3,i]*B3[8]+b[4,i]*B4[8]))/delta[8]+a2[1]*B1[8]+
a2[2]*B2[8]+a2[3]*B3[8]+a2[4]*B4[8]+b2[i]+beta[8,i])


}

   }
# Data
list(M=8,N =7,
      O1=structure(.Data=c(94.03,87.68,87.15,85.66,114.64,106.33,91.51,
90.67,97.74,93.63,85.18,109.08,119.29,101.42,
```

```
260.63,269.23,286.95,290.23,327.03,324.13,272.79,
137.51,139.75,130.87,130.86,150.21,155.2,149.59,
171.37,179.06,175.18,193.18,192.44,214.1,187.67,
174.42,184.09,161.06,175.28,183.91,202.62,178.61,
201.08,209.98,187.69,216.31,208.69,251.24,231.02,
185.59,191.62,172.37,195.77,183.91,231.54,205.2),.Dim=c(8,7)),

O2=structure(.Data=c(89.63 ,71.58 ,78.41 ,76.09 ,93.53 ,107.95 ,85.81,
87.17 ,77.23 ,82.49 ,80.21 ,97.31 ,116.19 ,89.40,
219.98 ,211.14 ,231.82 ,231.56 ,268.24 ,276.43 ,231.83,
123.12 ,117.91 ,113.18 ,116.39 ,133.72 ,138.47 ,139.20,
165.52 ,147.01 ,148.95 ,177.71 ,169.85 ,182.16 ,167.80,
166.18 ,157.69 ,141.87 ,159.98 ,167.09 ,178.98 ,163.22,
193.01 ,187.00 ,174.74 ,189.66 ,189.38 ,215.60 ,205.62,
178.56 ,174.48 ,162.66 ,176.22 ,172.77 ,202.04 ,189.26),.Dim=c(8,7)),

n1=structure(.Data=c(548,615,611,523,773,658,597,
496,595,584,482,655,668,599,
678,706,762,760,852,844,716,
553,586,568,534,631,633,598,
524,564,556,632,599,686,561,
537,593,504,561,571,646,528,
541,567,508,597,556,712,597,
516,529,471,556,511,692,551),.Dim=c(8,7)),


n2=structure(.Data=c(647,644,658,601,776,704,658,
596,616,607,554,712,691,614,
702,699,751,738,835,844,727,
593,598,599,593,668,631,679,
587,578,590,706,618,670,625,
594,631,545,630,610,671,603,
601,629,560,643,582,731,658,
568,602,544,627,568,739,627),.Dim=c(8,7)),

R=structure(.Data = c(1,0,0,0,0,1,0,0,0,0,1,0,0,0,0,1),.Dim = c(4,4)),



#with one knot at year 1997
    B1= c(0.000,0.618,0.490,0.238,0.061,0.022,0.001,0.000),
    B2= c(0.000,0.082,0.420,0.482,0.315,0.191,0.038,0.000),
    B3= c(0.000,0.003,0.090,0.271,0.499,0.521,0.331,0.000),
    B4= c(0.000,0.000,0.000,0.010,0.125,0.266,0.630,1.000),
```

```
num = c(2, 3, 2, 2, 2, 2, 1
),
adj = c(
7, 4,
6, 5, 3,
4, 2,
3, 1,
6, 2,
5, 2,
1
),
sumNumNeigh = 14)

#inits for 1997
list(alpha=c(-1,-0.5),a1=c(0.005,0.002,-1,-0.5),a2=c(0.005,0.002,1,1),tau.beta=10000,

omega=structure(.Data=c(5.5,1.5,-0.8,0.1,1.5,5.4,0.2,-0.8,-0.8,0.2,4.0,-2.0,0.1,-0.8,-2.0,8.2),.Dim = c(4,4)))

inits2
list(alpha=c(-1.1,-0.04),a1=c(0.1,0.7,0.4,0.8),a2=c(1,1,-1,-1),tau.beta=9000,tau.beta1=9000,tau.beta2=9000,
        b0=c(1,1,1,1,1,1,1),            b1=c(-0.1,-0.1,-0.2,-0.15,-0.17,-0.17,-0.13),
b2=c(-0.3,-0.6,-0.6,-0.5,-1.0,-0.9,-0.8),

                omega=structure(.Data = c(1,0,0,0,0,1,0,0,0,0,1,0,0,0,0,1),.Dim = c(4,4)))

)
```

Model 2

```
model
        {
             for(j in 1:M) {
                  for( i in 1:N) {
            O1[j,i]~ dbin(p1[j,i],n1[j,i])
                  O2[j,i]~ dbin(p2[j,i],n2[j,i])
            logit(p1[j,i]) <-alpha[1]+eta1[j,i,1]
                  logit(p2[j,i]) <-alpha[2]+eta2[j,i,2]
                    eta1[j,i,1]<-b0[i]*delta[j]+a1[1]*B1[j]+a1[2]*B2[j]+a1[3]*B3[j]+a1[4]*B4[j]+b1[i]

eta2[j,i,2]<-b0[i]/delta[j]+a2[1]*B1[j]+a2[2]*B2[j]+a2[3]*B3[j]+a2[4]*B4[j]+b2[i]+beta[j,i]
           }
         }
               # weights for the spatial adjacency matrix
                    for(k in 1: sumNumNeigh){
                          weights[k]<-1
                    }

        # Spatial priors

            for(j in 1:M) {
                  beta[j,1:N] ~ car.normal(adj[],weights[],num[],tau.beta)

                  }
        # other priors ,such as the intercepts,fixed effects,relative weight and variances
            alpha[1]~ dnorm(0.0,100)
                  OR.alpha[1]<-exp(alpha[1])
                  alpha[2]~ dnorm(0.0,1000)
                  OR.alpha[2]<-exp(alpha[2])

               sigma2.beta<-1/tau.beta

               tau.beta~dgamma(5,0.0005)

               for (P in 1:4){
                  a1[P]~ dnorm(0.0,100)
                  OR.a1[P]<-exp(a1[P])
                  a2[P]~ dnorm(0.0,1000)
                  OR.a2[P]<-exp(a2[P])
                    }
             for( i in 1:N) {
                    b0[i] ~ dnorm(0.0,100)
                    OR.b0[i]<-exp(b0[i])
```

```
            b1[i]    ~ dnorm(0.0,100)
            OR.b1[i]<-exp(b1[i])
            b2[i]    ~ dnorm(0.0,1000)
            OR.b2[i]<-exp(b2[i])

          }

      #scaling factor for relative strength of shared component for each disease
       for (j in 1:M){
          logdelta[j]~ dnorm(0.0,10)
          delta[j]<-exp(logdelta[j])
       #ratio(relative risk of disease 1 associated with shared component ) to disease 2 associated with shared component
           ORratio[j]<-pow(delta[j],2)
        }
#ouput
  for (i in 1:N){
    OR.beta1[i]<-exp(beta[1,i])
    OR.beta2[i]<-exp(beta[2,i])
    OR.beta3[i]<-exp(beta[3,i])
    OR.beta4[i]<-exp(beta[4,i])
    OR.beta5[i]<-exp(beta[5,i])
    OR.beta6[i]<-exp(beta[6,i])
    OR.beta7[i]<-exp(beta[7,i])
    OR.beta8[i]<-exp(beta[8,i])

    OR11[i]<-exp(b0[i]*delta[1]+a1[1]*B1[1]+a1[2]*B2[1]+a1[3]*B3[1]+a1[4]*B4[1]+b1[i])
    OR12[i]<-exp(b0[i]*delta[2]+a1[1]*B1[2]+a1[2]*B2[2]+a1[3]*B3[2]+a1[4]*B4[2]+b1[i])
    OR13[i]<-expb0[i]*delta[3]+a1[1]*B1[3]+a1[2]*B2[3]+a1[3]*B3[3]+a1[4]*B4[3]+b1[i])
    OR14[i]<-exp(b0[i]*delta[4]+a1[1]*B1[4]+a1[2]*B2[4]+a1[3]*B3[4]+a1[4]*B4[4]+b1[i])
    OR15[i]<-exp(b0[i]*delta[5]+a1[1]*B1[5]+a1[2]*B2[5]+a1[3]*B3[5]+a1[4]*B4[5]+b1[i])
    OR16[i]<-exp(b0[i]*delta[6]+a1[1]*B1[6]+a1[2]*B2[6]+a1[3]*B3[6]+a1[4]*B4[6]+b1[i])
    OR17[i]<-exp(b0[i]*delta[7]+a1[1]*B1[7]+a1[2]*B2[7]+a1[3]*B3[7]+a1[4]*B4[7]+b1[i])
    OR18[i]<-exp(b0[i]*delta[8]+a1[1]*B1[8]+a1[2]*B2[8]+a1[3]*B3[8]+a1[4]*B4[8]+b1[i])


OR21[i]<-exp(b0[i]/delta[1]+a2[1]*B1[1]+a2[2]*B2[1]+a2[3]*B3[1]+a2[4]*B4[1]+b2[i]+beta[1,i])

OR22[i]<-exp(b0[i]/delta[2]+a2[1]*B1[2]+a2[2]*B2[2]+a2[3]*B3[2]+a2[4]*B4[2]+b2[i]+beta[2,i])

OR23[i]<-exp(b0[i]/delta[3]+a2[1]*B1[3]+a2[2]*B2[3]+a2[3]*B3[3]+a2[4]*B4[3]+b2[i]+beta[3,i])

OR24[i]<-exp(b0[i]/delta[4]+a2[1]*B1[4]+a2[2]*B2[4]+a2[3]*B3[4]+a2[4]*B4[4]+b2[i]+beta[4,i])
```

```
OR25[i]<-exp(b0[i]/delta[5]+a2[1]*B1[5]+a2[2]*B2[5]+a2[3]*B3[5]+a2[4]*B4[5]+b2[i]+beta[5,i])

OR26[i]<-exp(b0[i]/delta[6]+a2[1]*B1[6]+a2[2]*B2[6]+a2[3]*B3[6]+a2[4]*B4[6]+b2[i]+beta[6,i])

OR27[i]<-exp(b0[i]/delta[7]+a2[1]*B1[7]+a2[2]*B2[7]+a2[3]*B3[7]+a2[4]*B4[7]+b2[i]+beta[7,i])

OR28[i]<-exp(b0[i]/delta[8]+a2[1]*B1[8]+a2[2]*B2[8]+a2[3]*B3[8]+a2[4]*B4[8]+b2[i]+beta[8,i])

}

    }
# Data
list(M=8,N =7,
        O1=structure(.Data=c(94.03,87.68,87.15,85.66,114.64,106.33,91.51,
90.67,97.74,93.63,85.18,109.08,119.29,101.42,
260.63,269.23,286.95,290.23,327.03,324.13,272.79,
137.51,139.75,130.87,130.86,150.21,155.2,149.59,
171.37,179.06,175.18,193.18,192.44,214.1,187.67,
174.42,184.09,161.06,175.28,183.91,202.62,178.61,
201.08,209.98,187.69,216.31,208.69,251.24,231.02,
185.59,191.62,172.37,195.77,183.91,231.54,205.2),.Dim=c(8,7)),

O2=structure(.Data=c(89.63 ,71.58 ,78.41 ,76.09 ,93.53 ,107.95 ,85.81,
87.17 ,77.23 ,82.49 ,80.21 ,97.31 ,116.19 ,89.40,
219.98 ,211.14 ,231.82 ,231.56 ,268.24 ,276.43 ,231.83,
123.12 ,117.91 ,113.18 ,116.39 ,133.72 ,138.47 ,139.20,
165.52 ,147.01 ,148.95 ,177.71 ,169.85 ,182.16 ,167.80,
166.18 ,157.69 ,141.87 ,159.98 ,167.09 ,178.98 ,163.22,
193.01 ,187.00 ,174.74 ,189.66 ,189.38 ,215.60 ,205.62,
178.56 ,174.48 ,162.66 ,176.22 ,172.77 ,202.04 ,189.26),.Dim=c(8,7)),

n1=structure(.Data=c(548,615,611,523,773,658,597,
496,595,584,482,655,668,599,
678,706,762,760,852,844,716,
553,586,568,534,631,633,598,
524,564,556,632,599,686,561,
537,593,504,561,571,646,528,
541,567,508,597,556,712,597,
516,529,471,556,511,692,551),.Dim=c(8,7)),


n2=structure(.Data=c(647,644,658,601,776,704,658,
596,616,607,554,712,691,614,
702,699,751,738,835,844,727,
```

```
593,598,599,593,668,631,679,
587,578,590,706,618,670,625,
594,631,545,630,610,671,603,
601,629,560,643,582,731,658,
568,602,544,627,568,739,627),.Dim=c(8,7)),

#with one knot at year 1997
    B1= c(0.000,0.618,0.490,0.238,0.061,0.022,0.001,0.000),
    B2= c(0.000,0.082,0.420,0.482,0.315,0.191,0.038,0.000),
    B3= c(0.000,0.003,0.090,0.271,0.499,0.521,0.331,0.000),
    B4= c(0.000,0.000,0.000,0.010,0.125,0.266,0.630,1.000),

num = c(2, 3, 2, 2, 2, 2, 1
),
adj = c(
7, 4,
6, 5, 3,
4, 2,
3, 1,
6, 2,
5, 2,
1
),
sumNumNeigh = 14)
#inits
list(alpha=c(0.4,-7.7),a1=c(-8.4,2.5,-1.6,-0.5),a2=c(10.2,0.4,5.7,4.4),tau.beta=50,
            b0=c(3.2,3.0,3.2,3.1,3.0,3.1,3.0),                          b1=c(-3.2,-3.5,-3.7,-3.5,-3.7,-3.9,-3.8),
b2=c(-0.3,-0.6,-0.6,-0.5,-1.0,-0.9,-0.8))

inits2
  list(alpha=c(-0.4,7.7),a1=c(8.4,-2.5,1.6,0.5),a2=c(-10.2,-0.4,-5.7,-4.4),tau.beta=10,
            b0=c(-3.2,-3.0,-3.2,-3.1,-3.0,-3.1,-3.0),                          b1=c(3.2,3.5,3.7,3.5,3.7,3.9,3.8),
b2=c(0.3,0.6,0.6,0.5,1.0,0.9,0.8))
```

Model 3

```
model
    {
        for(j in 1:M) {
            for( i in 1:N) {
        O1[j,i]~ dbin(p1[j,i],n1[j,i])
            O2[j,i]~ dbin(p2[j,i],n2[j,i])
        logit(p1[j,i]) <-alpha[1]+eta1[j,i,1]
            logit(p2[j,i]) <-alpha[2]+eta2[j,i,2]

eta1[j,i,1]<-(b0[i]+(b[1,i]*B1[j]+b[2,i]*B2[j]+b[3,i]*B3[j]+b[4,i]*B4[j]))*delta[j]+a1[1]*B1[j]+a1[2]*B2[j]+a1[3]*B3[j]+a1[4]*B4[j]+b1[i]

eta2[j,i,2]<-(b0[i]+(b[1,i]*B1[j]+b[2,i]*B2[j]+b[3,i]*B3[j]+b[4,i]*B4[j]))/delta[j]+a2[1]*B1[j]+a2[2]*B2[j]+a2[3]*B3[j]+a2[4]*B4[j]+b2[i]+beta[j,i]
        }
     }
        # weights for the spatial adjacency matrix
            for(k in 1: sumNumNeigh){
                weights[k]<-1
            }

    # Spatial priors
            b[1:4,1:N]~ mv.car(adj[],weights[],num[],omega[,])
              omega[1:4,1:4]~ dwish(R[,],4)

        for(j in 1:M) {
            beta[j,1:N] ~ car.normal(adj[],weights[],num[],tau.beta)

            }
    # other priors ,such as the intercepts,fixed effects,relative weight and variances
        alpha[1]~dnorm(0.0,100)
            OR.alpha[1]<-exp(alpha[1])
            alpha[2]~dnorm(0.0,1000)
            OR.alpha[2]<-exp(alpha[2])

        sigma2.beta<-1/tau.beta

        tau.beta~dgamma(5,0.0005)


        for (P in 1:4){
            a1[P]~dnorm(0.0,100)
            OR.a1[P]<-exp(a1[P])
```

```
            a2[P]~dnorm(0.0,1000)
            OR.a2[P]<-exp(a2[P])
              }
        for( i in 1:N) {
              b0[i] ~ dnorm(0.0,100)
              OR.b0[i]<-exp(b0[i])
              b1[i] ~ dnorm(0.0,100)
              OR.b1[i]<-exp(b1[i])
              b2[i] ~ dnorm(0.0,1000)
              OR.b2[i]<-exp(b2[i])

            }

    #scaling factor for relative strength of shared component for each disease
     for (j in 1:M){
           logdelta[j]~ dnorm(0.0,10)
           delta[j]<-exp(logdelta[j])
     #ratio(relative risk of disease 1 associated with shared component ) to disease 2 associated with shared component
             ORratio[j]<-pow(delta[j],2)
       }
#ouput
   for (i in 1:N){
      OR.beta1[i]<-exp(beta[1,i])
      OR.beta2[i]<-exp(beta[2,i])
      OR.beta3[i]<-exp(beta[3,i])
      OR.beta4[i]<-exp(beta[4,i])
      OR.beta5[i]<-exp(beta[5,i])
      OR.beta6[i]<-exp(beta[6,i])
      OR.beta7[i]<-exp(beta[7,i])
      OR.beta8[i]<-exp(beta[8,i])


OR11[i]<-exp((b0[i]+(b[1,i]*B1[1]+b[2,i]*B2[1]+b[3,i]*B3[1]+b[4,i]*B4[1]))*delta[1]+a1[1]*B1[1]+
a1[2]*B2[1]+a1[3]*B3[1]+a1[4]*B4[1]+b1[i])

OR12[i]<-exp( (b0[i]+(b[1,i]*B1[2]+b[2,i]*B2[2]+b[3,i]*B3[2]+b[4,i]*B4[2]))*delta[2]+a1[1]*B1[2]+
a1[2]*B2[2]+a1[3]*B3[2]+a1[4]*B4[2]+b1[i])

OR13[i]<-exp( (b0[i]+(b[1,i]*B1[3]+b[2,i]*B2[3]+b[3,i]*B3[3]+b[4,i]*B4[3]))*delta[3]+a1[1]*B1[3]+
a1[2]*B2[3]+a1[3]*B3[3]+a1[4]*B4[3]+b1[i])

OR14[i]<-exp( (b0[i]+(b[1,i]*B1[4]+b[2,i]*B2[4]+b[3,i]*B3[4]+b[4,i]*B4[4]))*delta[4]+a1[1]*B1[4]+
a1[2]*B2[4]+a1[3]*B3[4]+a1[4]*B4[4]+b1[i])
```

```
OR15[i]<-exp( (b0[i]+(b[1,i]*B1[5]+b[2,i]*B2[5]+b[3,i]*B3[5]+b[4,i]*B4[5]))*delta[5]+a1[1]*B1[5]+
a1[2]*B2[5]+a1[3]*B3[5]+a1[4]*B4[5]+b1[i])

OR16[i]<-exp( (b0[i]+(b[1,i]*B1[6]+b[2,i]*B2[6]+b[3,i]*B3[6]+b[4,i]*B4[6]))*delta[6]+a1[1]*B1[6]+
a1[2]*B2[6]+a1[3]*B3[6]+a1[4]*B4[6]+b1[i])

OR17[i]<-exp( (b0[i]+(b[1,i]*B1[7]+b[2,i]*B2[7]+b[3,i]*B3[7]+b[4,i]*B4[7]))*delta[7]+a1[1]*B1[7]+
a1[2]*B2[7]+a1[3]*B3[7]+a1[4]*B4[7]+b1[i])

OR18[i]<-exp( (b0[i]+(b[1,i]*B1[8]+b[2,i]*B2[8]+b[3,i]*B3[8]+b[4,i]*B4[8]))*delta[8]+a1[1]*B1[8]+
a1[2]*B2[8]+a1[3]*B3[8]+a1[4]*B4[8]+b1[i])


OR21[i]<-exp(  (b0[i]+(b[1,i]*B1[1]+b[2,i]*B2[1]+b[3,i]*B3[1]+b[4,i]*B4[1]))/delta[1]+a2[1]*B1[1]+
a2[2]*B2[1]+a2[3]*B3[1]+a2[4]*B4[1]+b2[i]+beta[1,i])

OR22[i]<-exp(  (b0[i]+(b[1,i]*B1[2]+b[2,i]*B2[2]+b[3,i]*B3[2]+b[4,i]*B4[2]))/delta[2]+a2[1]*B1[2]+
a2[2]*B2[2]+a2[3]*B3[2]+a2[4]*B4[2]+b2[i]+beta[2,i])

OR23[i]<-exp(  (b0[i]+(b[1,i]*B1[3]+b[2,i]*B2[3]+b[3,i]*B3[3]+b[4,i]*B4[3]))/delta[3]+a2[1]*B1[3]+
a2[2]*B2[3]+a2[3]*B3[3]+a2[4]*B4[3]+b2[i]+beta[3,i])

OR24[i]<-exp(  (b0[i]+(b[1,i]*B1[4]+b[2,i]*B2[4]+b[3,i]*B3[4]+b[4,i]*B4[4]))/delta[4]+a2[1]*B1[4]+
a2[2]*B2[4]+a2[3]*B3[4]+a2[4]*B4[4]+b2[i]+beta[4,i])

OR25[i]<-exp(  (b0[i]+(b[1,i]*B1[5]+b[2,i]*B2[5]+b[3,i]*B3[5]+b[4,i]*B4[5]))/delta[5]+a2[1]*B1[5]+
a2[2]*B2[5]+a2[3]*B3[5]+a2[4]*B4[5]+b2[i]+beta[5,i])

OR26[i]<-exp(  (b0[i]+(b[1,i]*B1[6]+b[2,i]*B2[6]+b[3,i]*B3[6]+b[4,i]*B4[6]))/delta[6]+a2[1]*B1[6]+
a2[2]*B2[6]+a2[3]*B3[6]+a2[4]*B4[6]+b2[i]+beta[6,i])

OR27[i]<-exp(  (b0[i]+(b[1,i]*B1[7]+b[2,i]*B2[7]+b[3,i]*B3[7]+b[4,i]*B4[7]))/delta[7]+a2[1]*B1[7]+
a2[2]*B2[7]+a2[3]*B3[7]+a2[4]*B4[7]+b2[i]+beta[7,i])

OR28[i]<-exp(  (b0[i]+(b[1,i]*B1[8]+b[2,i]*B2[8]+b[3,i]*B3[8]+b[4,i]*B4[8]))/delta[8]+a2[1]*B1[8]+
a2[2]*B2[8]+a2[3]*B3[8]+a2[4]*B4[8]+b2[i]+beta[8,i])

}

   }
# Data
list(M=8,N =7,
     O1=structure(.Data=c(94.03,87.68,87.15,85.66,114.64,106.33,91.51,
```

```
90.67,97.74,93.63,85.18,109.08,119.29,101.42,
260.63,269.23,286.95,290.23,327.03,324.13,272.79,
137.51,139.75,130.87,130.86,150.21,155.2,149.59,
171.37,179.06,175.18,193.18,192.44,214.1,187.67,
174.42,184.09,161.06,175.28,183.91,202.62,178.61,
201.08,209.98,187.69,216.31,208.69,251.24,231.02,
185.59,191.62,172.37,195.77,183.91,231.54,205.2),.Dim=c(8,7)),

O2=structure(.Data=c(89.63 ,71.58 ,78.41 ,76.09 ,93.53 ,107.95 ,85.81,
87.17 ,77.23 ,82.49 ,80.21 ,97.31 ,116.19 ,89.40,
219.98 ,211.14 ,231.82 ,231.56 ,268.24 ,276.43 ,231.83,
123.12 ,117.91 ,113.18 ,116.39 ,133.72 ,138.47 ,139.20,
165.52 ,147.01 ,148.95 ,177.71 ,169.85 ,182.16 ,167.80,
166.18 ,157.69 ,141.87 ,159.98 ,167.09 ,178.98 ,163.22,
193.01 ,187.00 ,174.74 ,189.66 ,189.38 ,215.60 ,205.62,
178.56 ,174.48 ,162.66 ,176.22 ,172.77 ,202.04 ,189.26),.Dim=c(8,7)),

n1=structure(.Data=c(548,615,611,523,773,658,597,
496,595,584,482,655,668,599,
678,706,762,760,852,844,716,
553,586,568,534,631,633,598,
524,564,556,632,599,686,561,
537,593,504,561,571,646,528,
541,567,508,597,556,712,597,
516,529,471,556,511,692,551),.Dim=c(8,7)),


n2=structure(.Data=c(647,644,658,601,776,704,658,
596,616,607,554,712,691,614,
702,699,751,738,835,844,727,
593,598,599,593,668,631,679,
587,578,590,706,618,670,625,
594,631,545,630,610,671,603,
601,629,560,643,582,731,658,
568,602,544,627,568,739,627),.Dim=c(8,7)),

R=structure(.Data = c(1,0,0,0,0,1,0,0,0,0,1,0,0,0,0,1),.Dim = c(4,4)),

#with one knot at year 2000
   B1= c(0.000,0.470,0.556,0.303,0.078,0.028,0.002,0.000),
   B2= c(0.000,0.057,0.347,0.495,0.371,0.232,0.048,0.000),
   B3= c(0.000,0.002,0.060,0.203,0.503,0.577,0.403,0.000),
   B4= c(0.000,0.000,0.000,0.000,0.048,0.162,0.548,1.000),
```

```
num = c(2, 3, 2, 2, 2, 2, 1
),
adj = c(
7, 4,
6, 5, 3,
4, 2,
3, 1,
6, 2,
5, 2,
1
),
sumNumNeigh = 14)
#inits for 2000
  # list(alpha=c(0.4,-7.7),a1=c(-8.4,2.5,-1.6,-0.5),a2=c(10.2,0.4,5.7,4.4),tau.beta=50,
        b0=c(3.2,3.0,3.2,3.1,3.0,3.1,3.0),                    b1=c(-3.2,-3.5,-3.7,-3.5,-3.7,-3.9,-3.8),
b2=c(-0.3,-0.6,-0.6,-0.5,-1.0,-0.9,-0.8),
              omega=structure(.Data                                                          =
c(6.8,1.7,-2.0,-0.8,1.7,4.6,0.6,-1.1,-2.0,0.6,3.5,-2.2,-0.8,-1.1,-2.2,7.5),.Dim = c(4,4)))

inits2
# list(alpha=c(-0.4,7.7),a1=c(8.4,-2.5,1.6,0.5),a2=c(-10.2,-0.4,-5.7,-4.4),tau.beta=10,
        b0=c(-3.2,-3.0,-3.2,-3.1,-3.0,-3.1,-3.0),                    b1=c(3.2,3.5,3.7,3.5,3.7,3.9,3.8),
b2=c(0.3,0.6,0.6,0.5,1.0,0.9,0.8),
                  omega=structure(.Data = c(1,0,0,0,0,1,0,0,0,0,1,0,0,0,0,1),.Dim = c(4,4)))
```

Model 4

```
model
        {
            for(j in 1:M) {
                for( i in 1:N) {
            O1[j,i]~ dbin(p1[j,i],n1[j,i])
                O2[j,i]~ dbin(p2[j,i],n2[j,i])
            logit(p1[j,i]) <-alpha[1]+eta1[j,i,1]
                logit(p2[j,i]) <-alpha[2]+eta2[j,i,2]
                eta1[j,i,1]<-b0[i]*delta[j]+a1[1]*B1[j]+a1[2]*B2[j]+a1[3]*B3[j]+a1[4]*B4[j]+b1[i]

eta2[j,i,2]<-b0[i]/delta[j]+a2[1]*B1[j]+a2[2]*B2[j]+a2[3]*B3[j]+a2[4]*B4[j]+b2[i]+beta[j,i]
          }
         }
              # weights for the spatial adjacency matrix
                 for(k in 1: sumNumNeigh){
                     weights[k]<-1
                 }

        # Spatial priors

                            for(j in 1:M) {
                beta[j,1:N] ~ car.normal(adj[],weights[],num[],tau.beta)

                }
        # other priors ,such as the intercepts,fixed effects,relative weight and variances
            alpha[1]~ dnorm(0.0,100)
                OR.alpha[1]<-exp(alpha[1])
                alpha[2]~ dnorm(0.0,1000)
                OR.alpha[2]<-exp(alpha[2])

              sigma2.beta<-1/tau.beta

              tau.beta~dgamma(5,0.0005)


              for (P in 1:4){
                a1[P]~ dnorm(0.0,100)
                OR.a1[P]<-exp(a1[P])
                a2[P]~ dnorm(0.0,1000)
                OR.a2[P]<-exp(a2[P])
                 }
            for( i in 1:N) {
                 b0[i] ~ dnorm(0.0,100)
```

```
                OR.b0[i]<-exp(b0[i])
                b1[i]    ~ dnorm(0.0,100)
                OR.b1[i]<-exp(b1[i])
                b2[i]    ~ dnorm(0.0,1000)
                OR.b2[i]<-exp(b2[i])

              }

      #scaling factor for relative strength of shared component for each disease
       for (j in 1:M){
            logdelta[j]~ dnorm(0.0,5.9)
            delta[j]<-exp(logdelta[j])
       #ratio(relative risk of disease 1 associated with shared component ) to disease 2 associated with shared component
             ORratio[j]<-pow(delta[j],2)
        }
#ouput
  for (i in 1:N){
    OR.beta1[i]<-exp(beta[1,i])
    OR.beta2[i]<-exp(beta[2,i])
    OR.beta3[i]<-exp(beta[3,i])
    OR.beta4[i]<-exp(beta[4,i])
    OR.beta5[i]<-exp(beta[5,i])
    OR.beta6[i]<-exp(beta[6,i])
    OR.beta7[i]<-exp(beta[7,i])
    OR.beta8[i]<-exp(beta[8,i])

    OR11[i]<-exp(b0[i]*delta[1]+a1[1]*B1[1]+a1[2]*B2[1]+a1[3]*B3[1]+a1[4]*B4[1]+b1[i])
    OR12[i]<-exp(b0[i]*delta[2]+a1[1]*B1[2]+a1[2]*B2[2]+a1[3]*B3[2]+a1[4]*B4[2]+b1[i])
    OR13[i]<-exp(b0[i]*delta[3]+a1[1]*B1[3]+a1[2]*B2[3]+a1[3]*B3[3]+a1[4]*B4[3]+b1[i])
    OR14[i]<-exp(b0[i]*delta[4]+a1[1]*B1[4]+a1[2]*B2[4]+a1[3]*B3[4]+a1[4]*B4[4]+b1[i])
    OR15[i]<-exp(b0[i]*delta[5]+a1[1]*B1[5]+a1[2]*B2[5]+a1[3]*B3[5]+a1[4]*B4[5]+b1[i])
    OR16[i]<-exp(b0[i]*delta[6]+a1[1]*B1[6]+a1[2]*B2[6]+a1[3]*B3[6]+a1[4]*B4[6]+b1[i])
    OR17[i]<-exp(b0[i]*delta[7]+a1[1]*B1[7]+a1[2]*B2[7]+a1[3]*B3[7]+a1[4]*B4[7]+b1[i])
    OR18[i]<-exp(b0[i]*delta[8]+a1[1]*B1[8]+a1[2]*B2[8]+a1[3]*B3[8]+a1[4]*B4[8]+b1[i])


OR21[i]<-exp(b0[i]/delta[1]+a2[1]*B1[1]+a2[2]*B2[1]+a2[3]*B3[1]+a2[4]*B4[1]+b2[i]+beta[1,i])

OR22[i]<-exp(b0[i]/delta[2]+a2[1]*B1[2]+a2[2]*B2[2]+a2[3]*B3[2]+a2[4]*B4[2]+b2[i]+beta[2,i])

OR23[i]<-exp(b0[i]/delta[3]+a2[1]*B1[3]+a2[2]*B2[3]+a2[3]*B3[3]+a2[4]*B4[3]+b2[i]+beta[3,i])

OR24[i]<-exp(b0[i]/delta[4]+a2[1]*B1[4]+a2[2]*B2[4]+a2[3]*B3[4]+a2[4]*B4[4]+b2[i]+beta[4,i])
```

```
OR25[i]<-exp(b0[i]/delta[5]+a2[1]*B1[5]+a2[2]*B2[5]+a2[3]*B3[5]+a2[4]*B4[5]+b2[i]+beta[5,i])

OR26[i]<-exp(b0[i]/delta[6]+a2[1]*B1[6]+a2[2]*B2[6]+a2[3]*B3[6]+a2[4]*B4[6]+b2[i]+beta[6,i])

OR27[i]<-exp(b0[i]/delta[7]+a2[1]*B1[7]+a2[2]*B2[7]+a2[3]*B3[7]+a2[4]*B4[7]+b2[i]+beta[7,i])

OR28[i]<-exp(b0[i]/delta[8]+a2[1]*B1[8]+a2[2]*B2[8]+a2[3]*B3[8]+a2[4]*B4[8]+b2[i]+beta[8,i])

}

   }
# Data
list(M=8,N =7,
      O1=structure(.Data=c(94.03,87.68,87.15,85.66,114.64,106.33,91.51
90.67,97.74,93.63,85.18,109.08,119.29,101.42
260.63,269.23,286.95,290.23,327.03,324.13,272.79
137.51,139.75,130.87,130.86,150.21,155.2,149.59
171.37,179.06,175.18,193.18,192.44,214.1,187.67
174.42,184.09,161.06,175.28,183.91,202.62,178.61
201.08,209.98,187.69,216.31,208.69,251.24,231.02
185.59,191.62,172.37,195.77,183.91,231.54,205.2),.Dim=c(8,7)),

O2=structure(.Data=c(89.63 ,71.58 ,78.41 ,76.09 ,93.53 ,107.95 ,85.81
87.17 ,77.23 ,82.49 ,80.21 ,97.31 ,116.19 ,89.40
219.98 ,211.14 ,231.82 ,231.56 ,268.24 ,276.43 ,231.83
123.12 ,117.91 ,113.18 ,116.39 ,133.72 ,138.47 ,139.20
165.52 ,147.01 ,148.95 ,177.71 ,169.85 ,182.16 ,167.80
166.18 ,157.69 ,141.87 ,159.98 ,167.09 ,178.98 ,163.22
193.01 ,187.00 ,174.74 ,189.66 ,189.38 ,215.60 ,205.62
178.56 ,174.48 ,162.66 ,176.22 ,172.77 ,202.04 ,189.26),.Dim=c(8,7)),

n1=structure(.Data=c(548,615,611,523,773,658,597,
496,595,584,482,655,668,599,
678,706,762,760,852,844,716,
553,586,568,534,631,633,598,
524,564,556,632,599,686,561,
537,593,504,561,571,646,528,
541,567,508,597,556,712,597,
516,529,471,556,511,692,551),.Dim=c(8,7)),


n2=structure(.Data=c(647,644,658,601,776,704,658,
596,616,607,554,712,691,614,
```

```
702,699,751,738,835,844,727,
593,598,599,593,668,631,679,
587,578,590,706,618,670,625,
594,631,545,630,610,671,603,
601,629,560,643,582,731,658,
568,602,544,627,568,739,627),.Dim=c(8,7)),



#with one knot at year 2000
    B1= c(0.000,0.470,0.556,0.303,0.078,0.028,0.002,0.000),
    B2= c(0.000,0.057,0.347,0.495,0.371,0.232,0.048,0.000),
    B3= c(0.000,0.002,0.060,0.203,0.503,0.577,0.403,0.000),
    B4= c(0.000,0.000,0.000,0.000,0.048,0.162,0.548,1.000),

num = c(2, 3, 2, 2, 2, 2, 1
),
adj = c(
7, 4,
6, 5, 3,
4, 2,
3, 1,
6, 2,
5, 2,
1
),
sumNumNeigh = 14)
#inits
list(alpha=c(0.4,-7.7),a1=c(-8.4,2.5,-1.6,-0.5),a2=c(10.2,0.4,5.7,4.4),tau.beta=50,
        b0=c(3.2,3.0,3.2,3.1,3.0,3.1,3.0),                b1=c(-3.2,-3.5,-3.7,-3.5,-3.7,-3.9,-3.8),
b2=c(-0.3,-0.6,-0.6,-0.5,-1.0,-0.9,-0.8))

inits2
  list(alpha=c(-0.4,7.7),a1=c(8.4,-2.5,1.6,0.5),a2=c(-10.2,-0.4,-5.7,-4.4),tau.beta=10,
        b0=c(-3.2,-3.0,-3.2,-3.1,-3.0,-3.1,-3.0),                b1=c(3.2,3.5,3.7,3.5,3.7,3.9,3.8),
b2=c(0.3,0.6,0.6,0.5,1.0,0.9,0.8))
```

Model 5 (The final model)

```
model
        {
             for(j in 1:M) {
                   for( i in 1:N) {
           O1[j,i]~ dbin(p1[j,i],n1[j,i])
                   O2[j,i]~ dbin(p2[j,i],n2[j,i])
           logit(p1[j,i]) <-alpha[1]+eta1[j,i,1]
                   logit(p2[j,i]) <-alpha[2]+eta2[j,i,2]

eta1[j,i,1]<-b0[i]*delta[j]+a1[1]*B1[j]+a1[2]*B2[j]+a1[3]*B3[j]+a1[4]*B4[j]+a1[5]*B5[j]+b1[i]

eta2[j,i,2]<-b0[i]/delta[j]+a2[1]*B1[j]+a2[2]*B2[j]+a2[3]*B3[j]+a2[4]*B4[j]+a2[5]*B5[j]+b2[i]+bet
a[j,i]
              }
            }
                 # weights for the spatial adjacency matrix
                     for(k in 1: sumNumNeigh){
                           weights[k]<-1
                     }

        # Spatial priors


                   for(j in 1:M) {
                   beta[j,1:N] ~ car.normal(adj[],weights[],num[],tau.beta)

                   }
        # other priors ,such as the intercepts,fixed effects,relative weight and variances
             alpha[1]~ dnorm(0.0,100)
                   OR.alpha[1]<-exp(alpha[1])
                   alpha[2]~ dnorm(0.0,1000)
                   OR.alpha[2]<-exp(alpha[2])
                 # prior 1
           sigma2.beta<-1/tau.beta
           tau.beta~dgamma(5,0.0005)
              #prior 2
                 #sigma.beta~dunif(0,1)
                # tau.beta<-1/(sigma.beta*sigma.beta)
              #prior 3
                # sigma2.beta~dnorm(0.0,100)I(0,)
                # tau.beta<-1/sigma2.beta
```

```
              for (P in 1:5){
                a1[P]~ dnorm(0.0,100)
                OR.a1[P]<-exp(a1[P])
                a2[P]~ dnorm(0.0,1000)
                OR.a2[P]<-exp(a2[P])
                 }
             for( i in 1:N) {
                  b0[i] ~ dnorm(0.0,100)
                  OR.b0[i]<-exp(b0[i])
                  b1[i]    ~ dnorm(0.0,100)
                  OR.b1[i]<-exp(b1[i])
                  b2[i]    ~ dnorm(0.0,1000)
                  OR.b2[i]<-exp(b2[i])

               }

      #scaling factor for relative strength of shared component for each disease
       for (j in 1:M){
             logdelta[j]~ dnorm(0.0,10)
             delta[j]<-exp(logdelta[j])
  #delta 1
          #      logdelta[j]~ dnorm(0.0,5.9)
            #      delta[j]<-exp(logdelta[j])
       #delta 2
          #    logdelta[j]~ dnorm(0.0,2.86)
          #    delta[j]<-exp(logdelta[j])
       #delta 3'
          #    logdelta[j]~ dunif(0.2,1.5)
            #      delta[j]<-exp(logdelta[j])
       # delta[j]~ dunif(0.2,5)
       #ratio(relative risk of disease 1 associated with shared component ) to disease 2
associated with shared component
              ORratio[j]<-pow(delta[j],2)
          }
#ouput
   for (i in 1:N){
       OR.beta1[i]<-exp(beta[1,i])
       OR.beta2[i]<-exp(beta[2,i])
       OR.beta3[i]<-exp(beta[3,i])
       OR.beta4[i]<-exp(beta[4,i])
       OR.beta5[i]<-exp(beta[5,i])
       OR.beta6[i]<-exp(beta[6,i])
       OR.beta7[i]<-exp(beta[7,i])
       OR.beta8[i]<-exp(beta[8,i])
```

```
OR11[i]<-exp(b0[i]*delta[1]+a1[1]*B1[1]+a1[2]*B2[1]+a1[3]*B3[1]+a1[4]*B4[1]+a1[5]*B5[1]+b1[
i])

OR12[i]<-exp(b0[i]*delta[2]+a1[1]*B1[2]+a1[2]*B2[2]+a1[3]*B3[2]+a1[4]*B4[2]+a1[5]*B5[2]+b1[
i])

OR13[i]<-exp(b0[i]*delta[3]+a1[1]*B1[3]+a1[2]*B2[3]+a1[3]*B3[3]+a1[4]*B4[3]+a1[5]*B5[3]+b1[
i])

OR14[i]<-exp(b0[i]*delta[4]+a1[1]*B1[4]+a1[2]*B2[4]+a1[3]*B3[4]+a1[4]*B4[4]+a1[5]*B5[4]+b1[
i])

OR15[i]<-exp(b0[i]*delta[5]+a1[1]*B1[5]+a1[2]*B2[5]+a1[3]*B3[5]+a1[4]*B4[5]+a1[5]*B5[5]+b1[
i])

OR16[i]<-exp(b0[i]*delta[6]+a1[1]*B1[6]+a1[2]*B2[6]+a1[3]*B3[6]+a1[4]*B4[6]+a1[5]*B5[6]+b1[
i])

OR17[i]<-exp(b0[i]*delta[7]+a1[1]*B1[7]+a1[2]*B2[7]+a1[3]*B3[7]+a1[4]*B4[7]+a1[5]*B5[7]+b1[
i])

OR18[i]<-exp(b0[i]*delta[8]+a1[1]*B1[8]+a1[2]*B2[8]+a1[3]*B3[8]+a1[4]*B4[8]+a1[5]*B5[8]+b1[
i])


OR21[i]<-exp(b0[i]/delta[1]+a2[1]*B1[1]+a2[2]*B2[1]+a2[3]*B3[1]+a2[4]*B4[1]+a2[5]*B5[1]+b2[i
]+beta[1,i])

OR22[i]<-exp(b0[i]/delta[2]+a2[1]*B1[2]+a2[2]*B2[2]+a2[3]*B3[2]+a2[4]*B4[2]+a2[5]*B5[2]+b2[i
]+beta[2,i])

OR23[i]<-exp(b0[i]/delta[3]+a2[1]*B1[3]+a2[2]*B2[3]+a2[3]*B3[3]+a2[4]*B4[3]+a2[5]*B5[3]+b2[i
]+beta[3,i])

OR24[i]<-exp(b0[i]/delta[4]+a2[1]*B1[4]+a2[2]*B2[4]+a2[3]*B3[4]+a2[4]*B4[4]+a2[5]*B5[4]+b2[i
]+beta[4,i])

OR25[i]<-exp(b0[i]/delta[5]+a2[1]*B1[5]+a2[2]*B2[5]+a2[3]*B3[5]+a2[4]*B4[5]+a2[5]*B5[5]+b2[i
]+beta[5,i])

OR26[i]<-exp(b0[i]/delta[6]+a2[1]*B1[6]+a2[2]*B2[6]+a2[3]*B3[6]+a2[4]*B4[6]+a2[5]*B5[6]+b2[i
]+beta[6,i])
```

```
OR27[i]<-exp(b0[i]/delta[7]+a2[1]*B1[7]+a2[2]*B2[7]+a2[3]*B3[7]+a2[4]*B4[7]+a2[5]*B5[7]+b2[i
]+beta[7,i])

OR28[i]<-exp(b0[i]/delta[8]+a2[1]*B1[8]+a2[2]*B2[8]+a2[3]*B3[8]+a2[4]*B4[8]+a2[5]*B5[8]+b2[i
]+beta[8,i])

}

    }
# Data
list(M=8,N =7,
        O1=structure(.Data=c(94.03,87.68,87.15,85.66,114.64,106.33,91.51,
90.67,97.74,93.63,85.18,109.08,119.29,101.42,
260.63,269.23,286.95,290.23,327.03,324.13,272.79,
137.51,139.75,130.87,130.86,150.21,155.2,149.59,
171.37,179.06,175.18,193.18,192.44,214.1,187.67,
174.42,184.09,161.06,175.28,183.91,202.62,178.61,
201.08,209.98,187.69,216.31,208.69,251.24,231.02,
185.59,191.62,172.37,195.77,183.91,231.54,205.2),.Dim=c(8,7)),

O2=structure(.Data=c(89.63 ,71.58 ,78.41 ,76.09 ,93.53 ,107.95 ,85.81,
87.17 ,77.23 ,82.49 ,80.21 ,97.31 ,116.19 ,89.40,
219.98 ,211.14 ,231.82 ,231.56 ,268.24 ,276.43 ,231.83,
123.12 ,117.91 ,113.18 ,116.39 ,133.72 ,138.47 ,139.20,
165.52 ,147.01 ,148.95 ,177.71 ,169.85 ,182.16 ,167.80,
166.18 ,157.69 ,141.87 ,159.98 ,167.09 ,178.98 ,163.22,
193.01 ,187.00 ,174.74 ,189.66 ,189.38 ,215.60 ,205.62,
178.56 ,174.48 ,162.66 ,176.22 ,172.77 ,202.04 ,189.26),.Dim=c(8,7)),

n1=structure(.Data=c(548,615,611,523,773,658,597,
496,595,584,482,655,668,599,
678,706,762,760,852,844,716,
553,586,568,534,631,633,598,
524,564,556,632,599,686,561,
537,593,504,561,571,646,528,
541,567,508,597,556,712,597,
516,529,471,556,511,692,551),.Dim=c(8,7)),


n2=structure(.Data=c(647,644,658,601,776,704,658,
596,616,607,554,712,691,614,
702,699,751,738,835,844,727,
593,598,599,593,668,631,679,
```

```
587,578,590,706,618,670,625,
594,631,545,630,610,671,603,
601,629,560,643,582,731,658,
568,602,544,627,568,739,627),.Dim=c(8,7)),



#with 2 knots at year 1997,2000
   B1= c(0.000,0.523,0.111,0.000,0.000,0.000,0.000,0.000),
   B2= c(0.000,0.174,0.689,0.432,0.111,0.041,0.003,0.000),
   B3= c(0.000,0.007,0.200,0.522,0.482,0.315,0.067,0.000),
   B4= c(0.000,0.000,0.000,0.046,0.359,0.482,0.383,0.000),
   B5= c(0.000,0.000,0.000,0.000,0.048,0.162,0.548,1.000),



num = c(2, 3, 2, 2, 2, 2, 1
),
adj = c(
7, 4,
6, 5, 3,
4, 2,
3, 1,
6, 2,
5, 2,
1
),
sumNumNeigh = 14)
#inits for 1997,2000
list(alpha=c(-1.1,0.0),a1=c(-0.4,0.9,0.0,0.8,0.7),a2=c(0.0,0.0,0.0,0.0,0.0),tau.beta=50,
          b0=c(-0.2,-0.3,-0.2,-0.2,-0.4,-0.3,-0.3),                    b1=c(0.0,-0.4,0.0,0.2,-0.5,-0.3,0.0),
b2=c(0.0,0.0,0.0,0.0,0.0,0.0,0.0))

inits2
  list(alpha=c(1.1,0.0),a1=c(0.4,-0.9,0.0,-0.8,-0.7),a2=c(0.0,0.0,0.0,0.0,0.0),tau.beta=10,
           b0=c(0.2,0.3,0.2,0.2,0.4,0.3,0.3),                            b1=c(0.0,0.4,0.0,-0.2,0.5,0.3,0.0),
b2=c(0.0,0.0,0.0,0.0,0.0,0.0,0.0))

#prior 2
#inits for 1997,2000
list(alpha=c(-1.1,0.0),a1=c(-0.4,0.9,0.0,0.8,0.7),a2=c(0.0,0.0,0.0,0.0,0.0),
          b0=c(-0.2,-0.3,-0.2,-0.2,-0.4,-0.3,-0.3),                    b1=c(0.0,-0.4,0.0,0.2,-0.5,-0.3,0.0),
b2=c(0.0,0.0,0.0,0.0,0.0,0.0,0.0))
```

inits2

```
list(alpha=c(1.1,0.0),a1=c(0.4,-0.9,0.0,-0.8,-0.7),a2=c(0.0,0.0,0.0,0.0,0.0),
        b0=c(0.2,0.3,0.2,0.2,0.4,0.3,0.3),                b1=c(0.0,0.4,0.0,-0.2,0.5,0.3,0.0),
b2=c(0.0,0.0,0.0,0.0,0.0,0.0,0.0))
```

Model 6

```
model
        {
            for(j in 1:M) {
                for( i in 1:N) {
            O1[j,i]~ dbin(p1[j,i],n1[j,i])
                O2[j,i]~ dbin(p2[j,i],n2[j,i])
            logit(p1[j,i]) <-alpha[1]+eta1[j,i,1]
                logit(p2[j,i]) <-alpha[2]+eta2[j,i,2]

eta1[j,i,1]<-b0[i]*delta[j]+a1[1]*B1[j]+a1[2]*B2[j]+a1[3]*B3[j]+a1[4]*B4[j]+a1[5]*B5[j]+b1[i]

eta2[j,i,2]<-b0[i]/delta[j]+a2[1]*B1[j]+a2[2]*B2[j]+a2[3]*B3[j]+a2[4]*B4[j]+a2[5]*B5[j]+b2[i]+bet
a[j,i]
         }
        }
              # weights for the spatial adjacency matrix
                 for(k in 1: sumNumNeigh){
                     weights[k]<-1
                 }

        # Spatial priors


                for(j in 1:M) {
                beta[j,1:N] ~ car.normal(adj[],weights[],num[],tau.beta)

                }
        # other priors ,such as the intercepts,fixed effects,relative weight and variances
            alpha[1]~ dnorm(0.0,100)
                OR.alpha[1]<-exp(alpha[1])
                alpha[2]~ dnorm(0.0,1000)
                OR.alpha[2]<-exp(alpha[2])

              sigma2.beta<-1/tau.beta

              tau.beta~dgamma(5,0.0005)


              for (P in 1:5){
                a1[P]~ dnorm(0.0,100)
                OR.a1[P]<-exp(a1[P])
                a2[P]~ dnorm(0.0,1000)
                OR.a2[P]<-exp(a2[P])
```

```
                }
            for( i in 1:N) {
                b0[i] ~ dnorm(0.0,100)
                OR.b0[i]<-exp(b0[i])
                b1[i]    ~ dnorm(0.0,100)
                OR.b1[i]<-exp(b1[i])
                b2[i]    ~ dnorm(0.0,1000)
                OR.b2[i]<-exp(b2[i])

              }

      #scaling factor for relative strength of shared component for each disease
        for (j in 1:M){
            logdelta[j]~ dnorm(0.0,5.9)
            delta[j]<-exp(logdelta[j])
        #ratio(relative risk of disease 1 associated with shared component ) to disease 2 
associated with shared component
             ORratio[j]<-pow(delta[j],2)
         }
#ouput
    for (i in 1:N){
        OR.beta1[i]<-exp(beta[1,i])
        OR.beta2[i]<-exp(beta[2,i])
        OR.beta3[i]<-exp(beta[3,i])
        OR.beta4[i]<-exp(beta[4,i])
        OR.beta5[i]<-exp(beta[5,i])
        OR.beta6[i]<-exp(beta[6,i])
        OR.beta7[i]<-exp(beta[7,i])
        OR.beta8[i]<-exp(beta[8,i])


OR11[i]<-exp(b0[i]*delta[1]+a1[1]*B1[1]+a1[2]*B2[1]+a1[3]*B3[1]+a1[4]*B4[1]+a1[5]*B5[1]+b1[
i])

OR12[i]<-exp(b0[i]*delta[2]+a1[1]*B1[2]+a1[2]*B2[2]+a1[3]*B3[2]+a1[4]*B4[2]+a1[5]*B5[2]+b1[
i])

OR13[i]<-exp(b0[i]*delta[3]+a1[1]*B1[3]+a1[2]*B2[3]+a1[3]*B3[3]+a1[4]*B4[3]+a1[5]*B5[3]+b1[
i])

OR14[i]<-exp(b0[i]*delta[4]+a1[1]*B1[4]+a1[2]*B2[4]+a1[3]*B3[4]+a1[4]*B4[4]+a1[5]*B5[4]+b1[
i])

OR15[i]<-exp(b0[i]*delta[5]+a1[1]*B1[5]+a1[2]*B2[5]+a1[3]*B3[5]+a1[4]*B4[5]+a1[5]*B5[5]+b1[
```

```
i])

OR16[i]<-exp(b0[i]*delta[6]+a1[1]*B1[6]+a1[2]*B2[6]+a1[3]*B3[6]+a1[4]*B4[6]+a1[5]*B5[6]+b1[
i])

OR17[i]<-exp(b0[i]*delta[7]+a1[1]*B1[7]+a1[2]*B2[7]+a1[3]*B3[7]+a1[4]*B4[7]+a1[5]*B5[7]+b1[
i])

OR18[i]<-exp(b0[i]*delta[8]+a1[1]*B1[8]+a1[2]*B2[8]+a1[3]*B3[8]+a1[4]*B4[8]+a1[5]*B5[8]+b1[
i])


OR21[i]<-exp(b0[i]/delta[1]+a2[1]*B1[1]+a2[2]*B2[1]+a2[3]*B3[1]+a2[4]*B4[1]+a2[5]*B5[1]+b2[i
]+beta[1,i])

OR22[i]<-exp(b0[i]/delta[2]+a2[1]*B1[2]+a2[2]*B2[2]+a2[3]*B3[2]+a2[4]*B4[2]+a2[5]*B5[2]+b2[i
]+beta[2,i])

OR23[i]<-exp(b0[i]/delta[3]+a2[1]*B1[3]+a2[2]*B2[3]+a2[3]*B3[3]+a2[4]*B4[3]+a2[5]*B5[3]+b2[i
]+beta[3,i])

OR24[i]<-exp(b0[i]/delta[4]+a2[1]*B1[4]+a2[2]*B2[4]+a2[3]*B3[4]+a2[4]*B4[4]+a2[5]*B5[4]+b2[i
]+beta[4,i])

OR25[i]<-exp(b0[i]/delta[5]+a2[1]*B1[5]+a2[2]*B2[5]+a2[3]*B3[5]+a2[4]*B4[5]+a2[5]*B5[5]+b2[i
]+beta[5,i])

OR26[i]<-exp(b0[i]/delta[6]+a2[1]*B1[6]+a2[2]*B2[6]+a2[3]*B3[6]+a2[4]*B4[6]+a2[5]*B5[6]+b2[i
]+beta[6,i])

OR27[i]<-exp(b0[i]/delta[7]+a2[1]*B1[7]+a2[2]*B2[7]+a2[3]*B3[7]+a2[4]*B4[7]+a2[5]*B5[7]+b2[i
]+beta[7,i])

OR28[i]<-exp(b0[i]/delta[8]+a2[1]*B1[8]+a2[2]*B2[8]+a2[3]*B3[8]+a2[4]*B4[8]+a2[5]*B5[8]+b2[i
]+beta[8,i])

}

    }
# Data
list(M=8,N =7,
        O1=structure(.Data=c(94.03,87.68,87.15,85.66,114.64,106.33,91.51,
90.67,97.74,93.63,85.18,109.08,119.29,101.42,
260.63,269.23,286.95,290.23,327.03,324.13,272.79,
```

```
137.51,139.75,130.87,130.86,150.21,155.2,149.59,
171.37,179.06,175.18,193.18,192.44,214.1,187.67,
174.42,184.09,161.06,175.28,183.91,202.62,178.61,
201.08,209.98,187.69,216.31,208.69,251.24,231.02,
185.59,191.62,172.37,195.77,183.91,231.54,205.2),.Dim=c(8,7)),

O2=structure(.Data=c(89.63 ,71.58 ,78.41 ,76.09 ,93.53 ,107.95 ,85.81,
87.17 ,77.23 ,82.49 ,80.21 ,97.31 ,116.19 ,89.40,
219.98 ,211.14 ,231.82 ,231.56 ,268.24 ,276.43 ,231.83,
123.12 ,117.91 ,113.18 ,116.39 ,133.72 ,138.47 ,139.20,
165.52 ,147.01 ,148.95 ,177.71 ,169.85 ,182.16 ,167.80,
166.18 ,157.69 ,141.87 ,159.98 ,167.09 ,178.98 ,163.22,
193.01 ,187.00 ,174.74 ,189.66 ,189.38 ,215.60 ,205.62,
178.56 ,174.48 ,162.66 ,176.22 ,172.77 ,202.04 ,189.26),.Dim=c(8,7)),

n1=structure(.Data=c(548,615,611,523,773,658,597,
496,595,584,482,655,668,599,
678,706,762,760,852,844,716,
553,586,568,534,631,633,598,
524,564,556,632,599,686,561,
537,593,504,561,571,646,528,
541,567,508,597,556,712,597,
516,529,471,556,511,692,551),.Dim=c(8,7)),


n2=structure(.Data=c(647,644,658,601,776,704,658,
596,616,607,554,712,691,614,
702,699,751,738,835,844,727,
593,598,599,593,668,631,679,
587,578,590,706,618,670,625,
594,631,545,630,610,671,603,
601,629,560,643,582,731,658,
568,602,544,627,568,739,627),.Dim=c(8,7)),


#with 2 knots at year 1997,2004
   B1= c(0.000,0.575,0.290,0.054,0.000,0.000,0.000,0.000),
   B2= c(0.000,0.124,0.572,0.525,0.175,0.064,0.004,0.000),
   B3= c(0.000,0.005,0.138,0.402,0.575,0.428,0.101,0.000),
   B4= c(0.000,0.000,0.000,0.020,0.250,0.485,0.531,0.000),
   B5= c(0.000,0.000,0.000,0.000,0.000,0.023,0.364,1.000),


num = c(2, 3, 2, 2, 2, 2, 1
```

```
),
adj = c(
7, 4,
6, 5, 3,
4, 2,
3, 1,
6, 2,
5, 2,
1
),
sumNumNeigh = 14)
#inits for 1997,2000
list(alpha=c(-1.1,0.0),a1=c(-0.4,0.9,0.0,0.8,0.7),a2=c(0.0,0.0,0.0,0.0,0.0),tau.beta=50,
       b0=c(-0.2,-0.3,-0.2,-0.2,-0.4,-0.3,-0.3),       b1=c(0.0,-0.4,0.0,0.2,-0.5,-0.3,0.0),
b2=c(0.0,0.0,0.0,0.0,0.0,0.0,0.0))

inits2
  list(alpha=c(1.1,0.0),a1=c(0.4,-0.9,0.0,-0.8,-0.7),a2=c(0.0,0.0,0.0,0.0,0.0),tau.beta=10,
        b0=c(0.2,0.3,0.2,0.2,0.4,0.3,0.3),       b1=c(0.0,0.4,0.0,-0.2,0.5,0.3,0.0),
b2=c(0.0,0.0,0.0,0.0,0.0,0.0,0.0))
```

Model 7

```
model
        {
            for(j in 1:M) {
                for( i in 1:N) {
            O1[j,i]~ dbin(p1[j,i],n1[j,i])
                O2[j,i]~ dbin(p2[j,i],n2[j,i])
            logit(p1[j,i]) <-alpha[1]+eta1[j,i,1]
                logit(p2[j,i]) <-alpha[2]+eta2[j,i,2]

eta1[j,i,1]<-b0[i]*delta[j]+a1[1]*B1[j]+a1[2]*B2[j]+a1[3]*B3[j]+a1[4]*B4[j]+a1[5]*B5[j]+a1[6]*B6[
j]+b1[i]

eta2[j,i,2]<-b0[i]/delta[j]+a2[1]*B1[j]+a2[2]*B2[j]+a2[3]*B3[j]+a2[4]*B4[j]+a2[5]*B5[j]+a2[6]*B6[
j]+b2[i]+beta[j,i]
            }
          }
              # weights for the spatial adjacency matrix
                  for(k in 1: sumNumNeigh){
                      weights[k]<-1
                  }

        # Spatial priors

                for(j in 1:M) {
                beta[j,1:N] ~ car.normal(adj[],weights[],num[],tau.beta)

                }
        # other priors ,such as the intercepts,fixed effects,relative weight and variances
            alpha[1]~ dnorm(0.0,100)
                OR.alpha[1]<-exp(alpha[1])
                alpha[2]~ dnorm(0.0,1000)
                OR.alpha[2]<-exp(alpha[2])
            # prior 1
          sigma2.beta<-1/tau.beta
            tau.beta~dgamma(0.1,0.001)
            #prior 2
              #         sigma.beta~dunif(0,1)
              #       tau.beta<-1/(sigma.beta*sigma.beta)
            #prior 3
              #    sigma2.beta~dnorm(0.0,100)I(0,)
                #    tau.beta<-1/sigma2.beta

              for (P in 1:6){
```

```
                  a1[P]~ dnorm(0.0,100)
                  OR.a1[P]<-exp(a1[P])
                  a2[P]~ dnorm(0.0,1000)
                  OR.a2[P]<-exp(a2[P])
                    }
              for( i in 1:N) {
                    b0[i] ~ dnorm(0.0,100)
                    OR.b0[i]<-exp(b0[i])
                    b1[i]    ~ dnorm(0.0,100)
                    OR.b1[i]<-exp(b1[i])
                    b2[i]    ~ dnorm(0.0,1000)
                    OR.b2[i]<-exp(b2[i])

                 }

        #scaling factor for relative strength of shared component for each disease
          for (j in 1:M){
          #delta 1
            #      logdelta[j]~ dnorm(0.0,5.9)
              #      delta[j]<-exp(logdelta[j])
          #delta 2
            #    logdelta[j]~ dnorm(0.0,2.86)
            #    delta[j]<-exp(logdelta[j])
          #delta 3'
            #    logdelta[j]~ dunif(0.2,1.5)
              #      delta[j]<-exp(logdelta[j])
            delta[j]~ dunif(0.2,5)
          #ratio(relative risk of disease 1 associated with shared component ) to disease 2
associated with shared component
                  ORratio[j]<-pow(delta[j],2)
            }
#ouput
    for (i in 1:N){
        OR.beta1[i]<-exp(beta[1,i])
        OR.beta2[i]<-exp(beta[2,i])
        OR.beta3[i]<-exp(beta[3,i])
        OR.beta4[i]<-exp(beta[4,i])
        OR.beta5[i]<-exp(beta[5,i])
        OR.beta6[i]<-exp(beta[6,i])
        OR.beta7[i]<-exp(beta[7,i])
        OR.beta8[i]<-exp(beta[8,i])


OR11[i]<-exp(b0[i]*delta[1]+a1[1]*B1[1]+a1[2]*B2[1]+a1[3]*B3[1]+a1[4]*B4[1]+a1[5]*B5[1]+a1[
```

```
6]*B6[1]+b1[i])

OR12[i]<-exp(b0[i]*delta[2]+a1[1]*B1[2]+a1[2]*B2[2]+a1[3]*B3[2]+a1[4]*B4[2]+a1[5]*B5[2]+a1[
6]*B6[2]+b1[i])

OR13[i]<-exp(b0[i]*delta[3]+a1[1]*B1[3]+a1[2]*B2[3]+a1[3]*B3[3]+a1[4]*B4[3]+a1[5]*B5[3]+a1[
6]*B6[3]+b1[i])

OR14[i]<-exp(b0[i]*delta[4]+a1[1]*B1[4]+a1[2]*B2[4]+a1[3]*B3[4]+a1[4]*B4[4]+a1[5]*B5[4]+a1[
6]*B6[4]+b1[i])

OR15[i]<-exp(b0[i]*delta[5]+a1[1]*B1[5]+a1[2]*B2[5]+a1[3]*B3[5]+a1[4]*B4[5]+a1[5]*B5[5]+a1[
6]*B6[5]+b1[i])

OR16[i]<-exp(b0[i]*delta[6]+a1[1]*B1[6]+a1[2]*B2[6]+a1[3]*B3[6]+a1[4]*B4[6]+a1[5]*B5[6]+a1[
6]*B6[6]+b1[i])

OR17[i]<-exp(b0[i]*delta[7]+a1[1]*B1[7]+a1[2]*B2[7]+a1[3]*B3[7]+a1[4]*B4[7]+a1[5]*B5[7]+a1[
6]*B6[7]+b1[i])

OR18[i]<-exp(b0[i]*delta[8]+a1[1]*B1[8]+a1[2]*B2[8]+a1[3]*B3[8]+a1[4]*B4[8]+a1[5]*B5[8]+a1[
6]*B6[8]+b1[i])


OR21[i]<-exp(b0[i]/delta[1]+a2[1]*B1[1]+a2[2]*B2[1]+a2[3]*B3[1]+a2[4]*B4[1]+a2[5]*B5[1]+a2[
6]*B6[1]+b2[i]+beta[1,i])

OR22[i]<-exp(b0[i]/delta[2]+a2[1]*B1[2]+a2[2]*B2[2]+a2[3]*B3[2]+a2[4]*B4[2]+a2[5]*B5[2]+a2[
6]*B6[2]+b2[i]+beta[2,i])

OR23[i]<-exp(b0[i]/delta[3]+a2[1]*B1[3]+a2[2]*B2[3]+a2[3]*B3[3]+a2[4]*B4[3]+a2[5]*B5[3]+a2[
6]*B6[3]+b2[i]+beta[3,i])

OR24[i]<-exp(b0[i]/delta[4]+a2[1]*B1[4]+a2[2]*B2[4]+a2[3]*B3[4]+a2[4]*B4[4]+a2[5]*B5[4]+a2[
6]*B6[4]+b2[i]+beta[4,i])

OR25[i]<-exp(b0[i]/delta[5]+a2[1]*B1[5]+a2[2]*B2[5]+a2[3]*B3[5]+a2[4]*B4[5]+a2[5]*B5[5]+a2[
6]*B6[5]+b2[i]+beta[5,i])

OR26[i]<-exp(b0[i]/delta[6]+a2[1]*B1[6]+a2[2]*B2[6]+a2[3]*B3[6]+a2[4]*B4[6]+a2[5]*B5[6]+a2[
6]*B6[6]+b2[i]+beta[6,i])

OR27[i]<-exp(b0[i]/delta[7]+a2[1]*B1[7]+a2[2]*B2[7]+a2[3]*B3[7]+a2[4]*B4[7]+a2[5]*B5[7]+a2[
6]*B6[7]+b2[i]+beta[7,i])
```

```
OR28[i]<-exp(b0[i]/delta[8]+a2[1]*B1[8]+a2[2]*B2[8]+a2[3]*B3[8]+a2[4]*B4[8]+a2[5]*B5[8]+a2[
6]*B6[8]+b2[i]+beta[8,i])

}

    }
# Data
list(M=8,N =7,
        O1=structure(.Data=c(94.03,87.68,87.15,85.66,114.64,106.33,91.51,
90.67,97.74,93.63,85.18,109.08,119.29,101.42,
260.63,269.23,286.95,290.23,327.03,324.13,272.79,
137.51,139.75,130.87,130.86,150.21,155.2,149.59,
171.37,179.06,175.18,193.18,192.44,214.1,187.67,
174.42,184.09,161.06,175.28,183.91,202.62,178.61,
201.08,209.98,187.69,216.31,208.69,251.24,231.02,
185.59,191.62,172.37,195.77,183.91,231.54,205.2),.Dim=c(8,7)),

O2=structure(.Data=c(89.63 ,71.58 ,78.41 ,76.09 ,93.53 ,107.95 ,85.81,
87.17 ,77.23 ,82.49 ,80.21 ,97.31 ,116.19 ,89.40,
219.98 ,211.14 ,231.82 ,231.56 ,268.24 ,276.43 ,231.83,
123.12 ,117.91 ,113.18 ,116.39 ,133.72 ,138.47 ,139.20,
165.52 ,147.01 ,148.95 ,177.71 ,169.85 ,182.16 ,167.80,
166.18 ,157.69 ,141.87 ,159.98 ,167.09 ,178.98 ,163.22,
193.01 ,187.00 ,174.74 ,189.66 ,189.38 ,215.60 ,205.62,
178.56 ,174.48 ,162.66 ,176.22 ,172.77 ,202.04 ,189.26),.Dim=c(8,7)),

n1=structure(.Data=c(548,615,611,523,773,658,597,
496,595,584,482,655,668,599,
678,706,762,760,852,844,716,
553,586,568,534,631,633,598,
524,564,556,632,599,686,561,
537,593,504,561,571,646,528,
541,567,508,597,556,712,597,
516,529,471,556,511,692,551),.Dim=c(8,7)),


n2=structure(.Data=c(647,644,658,601,776,704,658,
596,616,607,554,712,691,614,
702,699,751,738,835,844,727,
593,598,599,593,668,631,679,
587,578,590,706,618,670,625,
594,631,545,630,610,671,603,
601,629,560,643,582,731,658,
```

```
568,602,544,627,568,739,627),.Dim=c(8,7)),



#with three knots at year 1997,2000,2004
   B1= c(0.000,0.523,0.111,0.000,0.000,0.000,0.000,0.000),
   B2= c(0.000,0.170,0.581,0.176,0.000,0.000,0.000,0.000),
   B3= c(0.000,0.011,0.308,0.732,0.318,0.116,0.007,0.000),
   B4= c(0.000,0.000,0.000,0.092,0.550,0.479,0.124,0.000),
   B5= c(0.000,0.000,0.000,0.000,0.132,0.382,0.504,0.000),
   B6= c(0.000,0.000,0.000,0.000,0.000,0.023,0.364,1.000),


num = c(2, 3, 2, 2, 2, 2, 1
),
adj = c(
7, 4,
6, 5, 3,
4, 2,
3, 1,
6, 2,
5, 2,
1
),
sumNumNeigh = 14)
#inits for prior 1 and prior2
list(alpha=c(-1.1,0.0),a1=c(-0.5,1.1,0.1,0.5,0.6,0.7),a2=c(0.0,0.0,0.0,0.0,0.0,0.0),tau.beta=45,
        b0=c(-0.2,-0.3,-0.2,-0.2,-0.3,-0.3,-0.2),            b1=c(0.0,-0.4,-0.1,0.2,-0.5,-0.3,0.0),
b2=c(0.0,0.0,0.0,0.0,0.0,0.0,0.0))

inits2
 list(alpha=c(1.1,0.0),a1=c(0.5,-1.1,-0.1,-0.5,-0.6,-0.7),a2=c(0.0,0.0,0.0,0.0,0.0,0.0),tau.beta=10,
        b0=c(0.2,0.3,0.2,0.2,0.3,0.3,0.2),               b1=c(0.0,0.4,0.1,-0.2,0.5,0.3,0.0),
b2=c(0.0,0.0,0.0,0.0,0.0,0.0,0.0))
```
