# Supplementary figures and images for: Application of SCM with Bayesian B-Spline to Spatio-Temporal Analysis of Hypertension in China
Source: Int J Environ Res Public Health. 2018 Jan 2;15(1):55. doi: 10.3390/ijerph15010055 (PMC5800154; doi:10.3390/ijerph15010055)

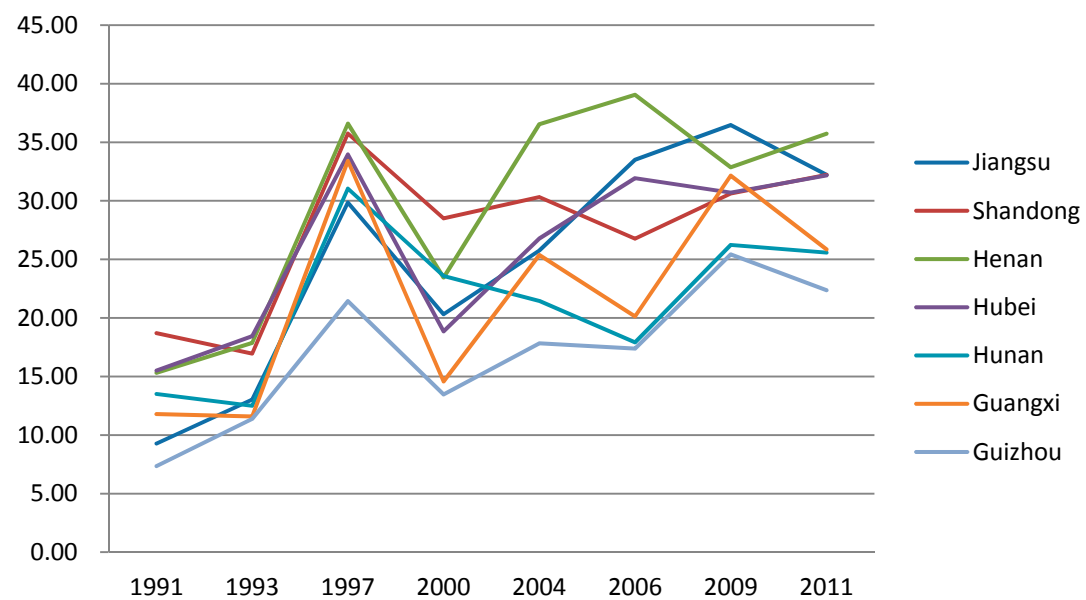

**Figure 1.** Prevalence of hypertension during eight waves surveys of each region

Supplement: Supplementary file 1 [file ijerph-15-00055-s001.zip › supplementary2.pdf]
